# Soft, Deformable Polyurethane-Boronic Acid Nanoparticles as Dynamic Cross-Linkers to Construct 3D-Bioprintable Hydrogels

**DOI:** 10.1021/acsami.5c07208

**Published:** 2025-07-08

**Authors:** Yung-Chen Chang, Tsai-Yu Chen, Yi-Ming Sun, Shan-hui Hsu

**Affiliations:** † Institute of Polymer Science and Engineering, 33561 National Taiwan University, Taipei 106319, Taiwan, Republic of China; ‡ Department of Chemical Engineering and Materials Science, 34895 Yuan Ze University, Chung-Li, Taoyuan 32003, Taiwan, Republic of China

**Keywords:** 3D printing, nanoparticles, glucose-sensitive hydrogel, polyurethane, small-angle X-ray scattering

## Abstract

Addition of nanoparticles in a hydrogel can enhance its three-dimensional (3D) printability. However, the role of soft, deformable nanoparticles in the 3D printability of a hydrogel network has not been explored so far. In this study, two boronic acid-functionalized polyurethane (PU) nanoparticles PUB and PUB′ are synthesized as soft dynamic nanocross-linkers to construct a 3D bioprintable hydrogel. The soft segment of PUB consists of poly­(ε-caprolactone) (PCL) solely while that of PUB′ consists of PCL, poly­(d,l-lactide), and poly­(3-hydroxybutyrate) in a 0.7/0.2/0.1 molar ratio. Small-angle X-ray scattering (SAXS) reveals that PUB nanoparticles are nearly spherical while PUB′ nanoparticles are ellipsoidal. A PUB′-cross-linked poly­(ethylene glycol) hydrogel based on dynamic click chemistry has greater shear modulus and creep resistance than a PUB-cross-linked hydrogel. When printed through a small (160 μm) nozzle, the PUB′-based hydrogel exhibits superior stackability and filament resolution. Time-resolved SAXS analysis unveils that PUB′ nanoparticles elongate and maintain a stable ellipsoidal morphology in the network during gelation, contributing to a higher packing density (particle volume fraction 38%) and 3D stackability of the hydrogel. Meanwhile, PUB nanoparticles transform from spherical to ellipsoidal and are eventually flattened, leading to a low packing density (particle volume fraction 18%) of the hydrogel. Moreover, endothelial cells laden in both hydrogels show high vitality (∼92%). The unique shape deformation phenomenon of the PU-boronic acid nanocross-linker during gelation and the resulted high-density packing in the dynamic network provide insights into the role of soft nanoparticle morphology in the stackability of a dynamic self-healing hydrogel and the role of particle packing in designing 3D hydrogel inks.

## Graphical abstract

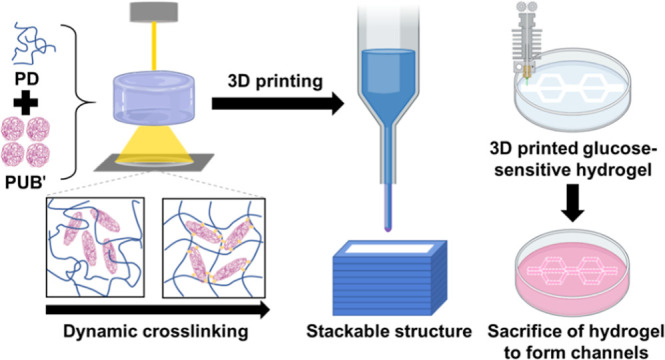

## 1 Introduction

Hydrogels are three-dimensional (3D) cross-linked polymer networks, characterized by high water retention capability and excellent elasticity. In recent years, they have found extensive applications in the biomedical field.^1^ Among them, smart hydrogels such as self-healing hydrogels,^2^ injectable hydrogels,^3^ and environmentally responsive hydrogels^4^ have garnered attention as potential biomaterials due to their exceptional functionalities. Self-healing hydrogels can utilize dynamic covalent bonds and noncovalent interactions, including boronate ester bonds, imine bonds, hydrogen bonds, or host–guest interactions. Injectable hydrogels offer in situ formability, allowing minimally invasive surgical procedures to be performed.^5^ Meanwhile, environmentally responsive hydrogels can change their chemical properties or physical structures in response to environmental stimuli and are commonly used for drug delivery.^6^ Sacrificial hydrogels are a subtype of environmentally responsive hydrogels, notable for their controlled dissolution or degradation to facilitate structural formation or modification.^7^ This property makes sacrificial hydrogels particularly valuable in the field of tissue engineering, such as for creating vascularized channels.^8^

Sacrificial hydrogels are materials that can be selectively removed or degraded from composite structures, leaving behind intricate and well-defined patterns or matrices.^9^ Sacrificial hydrogels have attracted research interest for their potential to address complex challenges in constructing vascularized channels for tissue engineering. In literature, multiple materials such as carbohydrate glass,^10^ gelatin,^11^ and Pluronic-F127 ^12^ have been explored as sacrificial materials for 3D printing to construct vascular channels within tissue scaffolds. However, the conventional removal of these sacrificial templates often necessitates meticulous temperature control or the incorporation of solvents, which may inflict damage upon the nonsacrificial hydrogel or pose potential harm to residing cells. A novel sacrificial hydrogel with glucose sensitivity was previously developed, which contains borax, which has the capability to detect various types of sugars.^13^ This glucose-sensitive sacrificial hydrogel offers the advantage of rapid dissolution in culture media and better preservation of the integrity of microchannel structures.^14^ Meanwhile, nearly all sacrificial hydrogels containing boronic acid show cytotoxicity and have rather short gelation times due to the fast formation of boronate ester bonds, which causes difficulty in 3D printing. Although a new glucose-sensitive sacrificial hydrogel incorporating polyethyleneimine (PEI) into borax has successfully slowed down the gelation rate and minimized cytotoxicity, the printing has to be performed by the gel-in-gel strategy to prevent collapse in higher layers.^15^

Polyurethane (PU) as a versatile polymeric material is widely employed across various industries owing to its exceptional performance.^16–18^ PU comprising both soft and hard segments exhibits microphase separation, which provides PU with flexibility and tunable mechanical characteristics.^19^ The waterborne biodegradable PU nanoparticles, distinguished by its biocompatibility and environmental benefits compared to solvent-borne PU, are utilized in various biomedical applications.^20^ Particularly, the waterborne biodegradable PU nanoparticles incorporating oligodiols such as poly­(ε-caprolactone) (PCL), polylactide (PLA), and poly­(3-hydroxybutyrate) (PHB) diols as soft segments has the ability to enhance cell adhesion, proliferation, and differentiation.^21–23^ Moreover, the PCL–PLA-based waterborne PU undergoes sol–gel transition at 37 °C, which enables its application as a bioink for printing scaffolds along with cells near the human body temperature.^24^ Nevertheless, relying solely on PU as a bioink component might lead to challenges such as low resolution and poor stackability. To overcome these issues, a combination of PU and gelatin was utilized to achieve higher resolution and stacking properties. The PU–gelatin composite bioink, capable of passing through an 80 μm nozzle, can be stacked up to 80 layers.^25^ Nevertheless, due to poor gelling of gelatin at 37 °C, a precise temperature control is essential during printing.^26^ A modified bioink comprising PU, glycol chitosan, and polyethylene oxide capped with benzaldehyde is unaffected by temperature fluctuations and can pass through a 160 μm nozzle with consistent stacking.^26^ These PU nanocomposite bioinks often possess good stackbabiliy, consistent with the literature of nanocomposite bioinks constructed from a chemical network with silicate nanoparticles,^27,28^ with the difference being that PU nanoparticles are biodegradable and rather soft.

Although the PU and PU nanocomposite bioink systems exhibit good biocompatibility and printing performance, the PU bioinks do not have much environmental responsiveness. The insufficient stimulus-responsive properties restrict the application of PU bioink as an environmentally sensitive sacrificial hydrogel. Introducing borax into a side chain endows a polymer with glucose sensitivity for serving as a sacrificial hydrogel.^13^ However, conventional glucose-sensitive hydrogels often suffer from rapid gelation, discontinuous extrusion, and needle clogging, leading to poor stacking performance. As a result, they typically rely on gel-in-gel printing methods, which significantly compromise the printing resolution and limit the fabrication of highly precise structures. To address these challenges, we propose that incorporating soft PU nanocross-linkers—with tunable soft segment compositions—into the hydrogel network can enhance injectability, delay premature gelation, and extend the working window. Furthermore, the deformable structural properties and dynamic network interactions enabled by different PU compositions are expected to improve the structural stability of printed constructs. In this study, novel borax-containing PU nanoparticles (PUB and PUB′) were synthesized by replacing one of the chain extenders ethylenediamine (EDA) with borax. These PUB and PUB′ nanoparticles can cross-link diol-containing polymers through reversible click reaction to generate dynamic boronate ester bonding. The diol-containing polymer selected for the study was a polyethylene glycol diacrylate (PEGDA)-dithiothreitol (DTT) polymer, which can react with a soft PUB or PUB′ nanocross-linker to form a glucose-sensitive hydrogel. Interestingly, the dynamic cross-linking process of PUB or PUB′ with the main chain induces varying degrees of nanocross-linker deformation during gelation, which impacts the rheological properties of the hydrogel and further modifies its 3D-printing performance. Moreover, the PUB- and PUB′-based glucose-sensitive hydrogel offers an extended gelation time, which significantly expands the working window during the printing process. The new PU-based glucose-sensitive hydrogels not only maintain cell survival but also ensure the precision of the bioprinted filaments, which open a new avenue for applying PU bioinks in advanced tissue engineering. Particularly, these innovative PU nanocross-linkers and the unique phenomenon of particle shape deformation during dynamic cross-linking offer novel insights and perspectives for designing composite bioinks with tunable gelling kinetics using soft nanocross-linkers.

## 2 Results

### 2.1 Synthesis of Waterborne Biodegradable Boronic Acid-Functionalized PU (PUB and PUB′) as Novel Cross-Linkers

The water dispersion of biodegradable PUB based on the PCL soft segment was synthesized by replacing one of the chain extenders (i.e., EDA) of the system with borax. The synthetic route and chemical composition of PUB, as compared to those of NH_2_-capped PU, are depicted in Figure 1A. The hydrodynamic radius (*R*_h_), zeta potential, polydispersity index (PDI), and average molecular weight (*M*_w_) of the PUB and the PU nanoparticles are listed in Tables 1 and S1, respectively. The *R*_h_ of PUB determined by dynamic light scattering (DLS) was 19.3 ± 4.6 nm, which exceeded that of PU (14.3 ± 3.5 nm). Meanwhile, the zeta potential of the PUB dispersion was −44.7 ± 1.56 mV, which was a more negative value compared to that of PU (−40.2 ± 1.21 mV). These results suggested that the suspended PUB nanoparticles were as stable as the PU nanoparticles. The PDI values of PUB and PU nanoparticles in dispersion were 0.055 and 0.059, respectively, where PDI values less than 1 refer to a uniform size distribution. The *M*_w_ values of PUB and PU measured by gel permeation chromatography (GPC) were about 70 and 91 kDa, respectively. The Mw of PU was relatively higher due to more chain extension by EDA.

**Figure 1:**
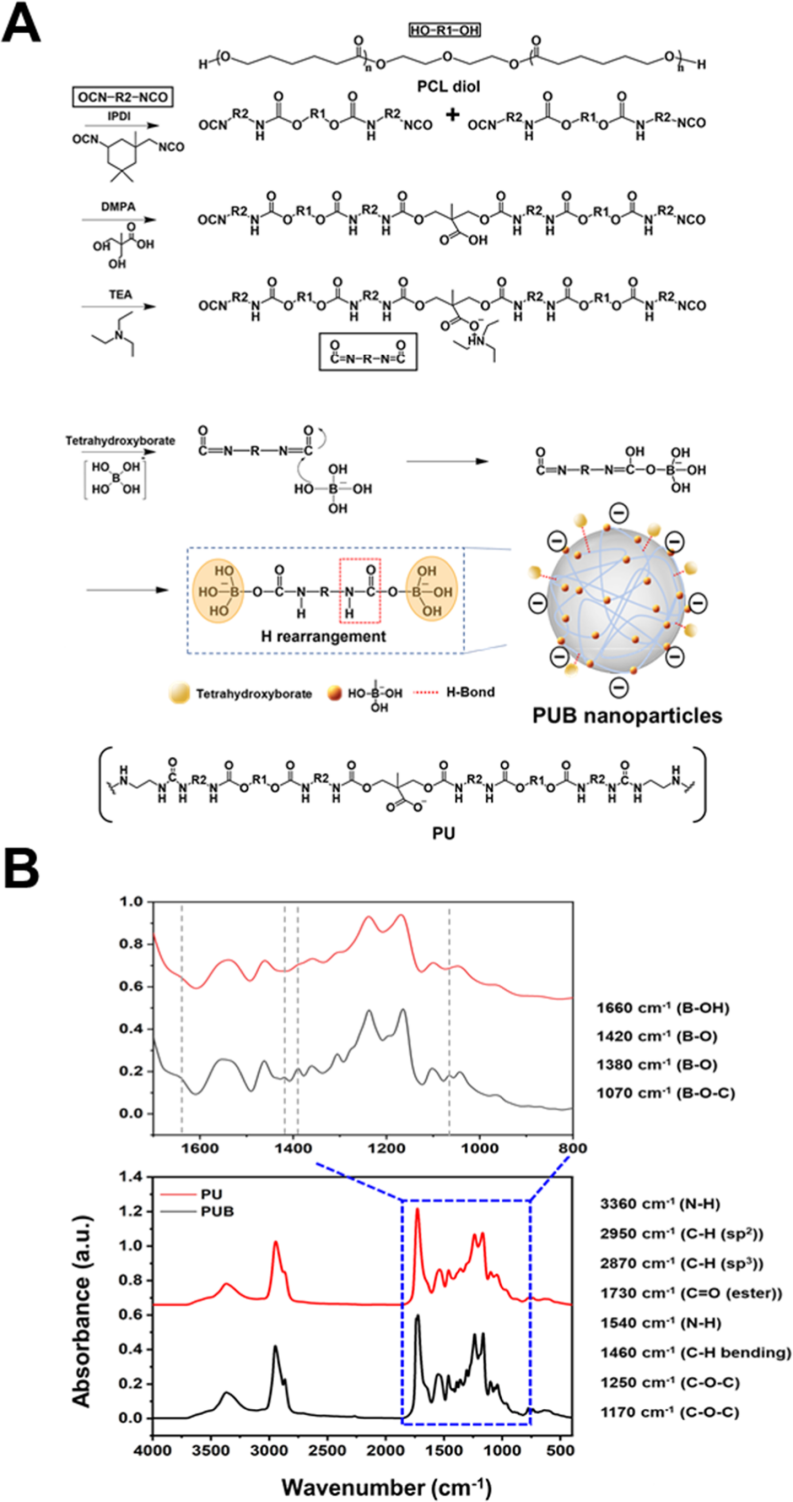
Synthesis of nanocross-linker PUB. (A) Synthesis scheme for waterborne biodegradable PUB. (B) FTIR spectra of PUB and PU.

**Table 1:** Hydrodynamic Size, Radius of Gyration, Shape Factor, Zeta Potential, and PDI of PUB and PUB′ Nanoparticles in a Water Suspension, along with the *M*_w_ of PUB and PUB′ Chains (Measured after Dissolution in DMAc)

	*R*_h_ (nm)	*R*_g_ (nm)	*R*_g_/*R*_h_ (shape factor)	zeta potential (mV)	PDI	*M*_w_
PUB	19.3 ± 4.6	18.6 ± 1.5	0.96	–44.7 ± 1.56	∼0.055	73,262
PUB′	12.4 ± 2.8	13.7 ± 1.6	1.10	–46.3 ± 1.82	∼0.051	75,850

Fourier transform infrared (FTIR) spectroscopy analysis was employed to confirm the successful incorporation of tetrahydroxyborate onto the PU chain. The spectra are shown in Figure 1B. The peak observed at 3360 cm^–1^ corresponds to the N–H stretching vibrations, while those observed at 2950 cm^–1^ and 2870 cm^–1^ indicate CH (sp^2^) stretching and CH (sp^3^) stretching, respectively. The absence of an absorption peak at 2260 cm^–1^ (NCO) in both the PUB and the PU spectra indicates that the reaction has proceeded to completion. Furthermore, the FTIR spectra of PU and PUB both exhibited characteristic bending vibration peaks at 1730 cm^–1^ (C=O), 1540 cm^–1^ (N–H), and 1460 cm^–1^ (C–H). The peaks at 1250 cm^–1^ and 1170 cm^–1^ were identified as characteristic of the C–O–C functional group. These peaks are representative of the PU backbone. In PUB, emerging peaks were observed at 1660 cm^–1^ (B–OH), 1420 and 1380 cm^–1^ (B–O), and 1070 cm^–1^ (B–O–C).^29^ These results substantiate the occurrence of a reaction between the isocyanate groups and the tetrahydroxyborate. However, the efficiency of covalent bond formation during nanoparticle synthesis may be kinetically constrained by rapid hydrolysis of isocyanate (−NCO) groups in aqueous media. Accordingly, we propose that the borax–PU interaction during PUB formation likely involves both covalent bonding and noncovalent adsorption. The former arises from partial B–O–C linkages on the PU backbone, while the latter results from hydrogen bonding between borate species and nanoparticle surfaces. These combined interactions stabilize the nanoparticle architecture and enable reversible boronate bonding with diol-containing PD chains. The chemical composition of PUB in the current study can be further adjusted by changing the soft segment component, which may modify the rheological property and cross-linking kinetics of PU–boronic acid nanoparticles. Specifically, PUB′ based on a more complicated ternary soft segment composition was synthesized (Figure 2A). The ternary PUB′ contained PDLLA and PHB segments, in addition to PCL segments. The *R*_h_, zeta potential, PDI, and *M*_w_ of PUB′ nanoparticles in comparison to those of PUB are shown in Table 1. The *R*_h_ of PUB′ was 12.4 ± 2.8 nm, with a surface zeta potential of −46.3 ± 1.82 mV. These results suggest that PUB′ remains stably suspended in water. The PDI of PUB′ in dispersion was measured at 0.051. The *M*_w_ of PUB′ was approximately 70 kDa, not significantly different from that of PUB. Meanwhile, the FTIR spectra of PUB′ and PUB showed comparable features (Figure 2B).

**Figure 2:**
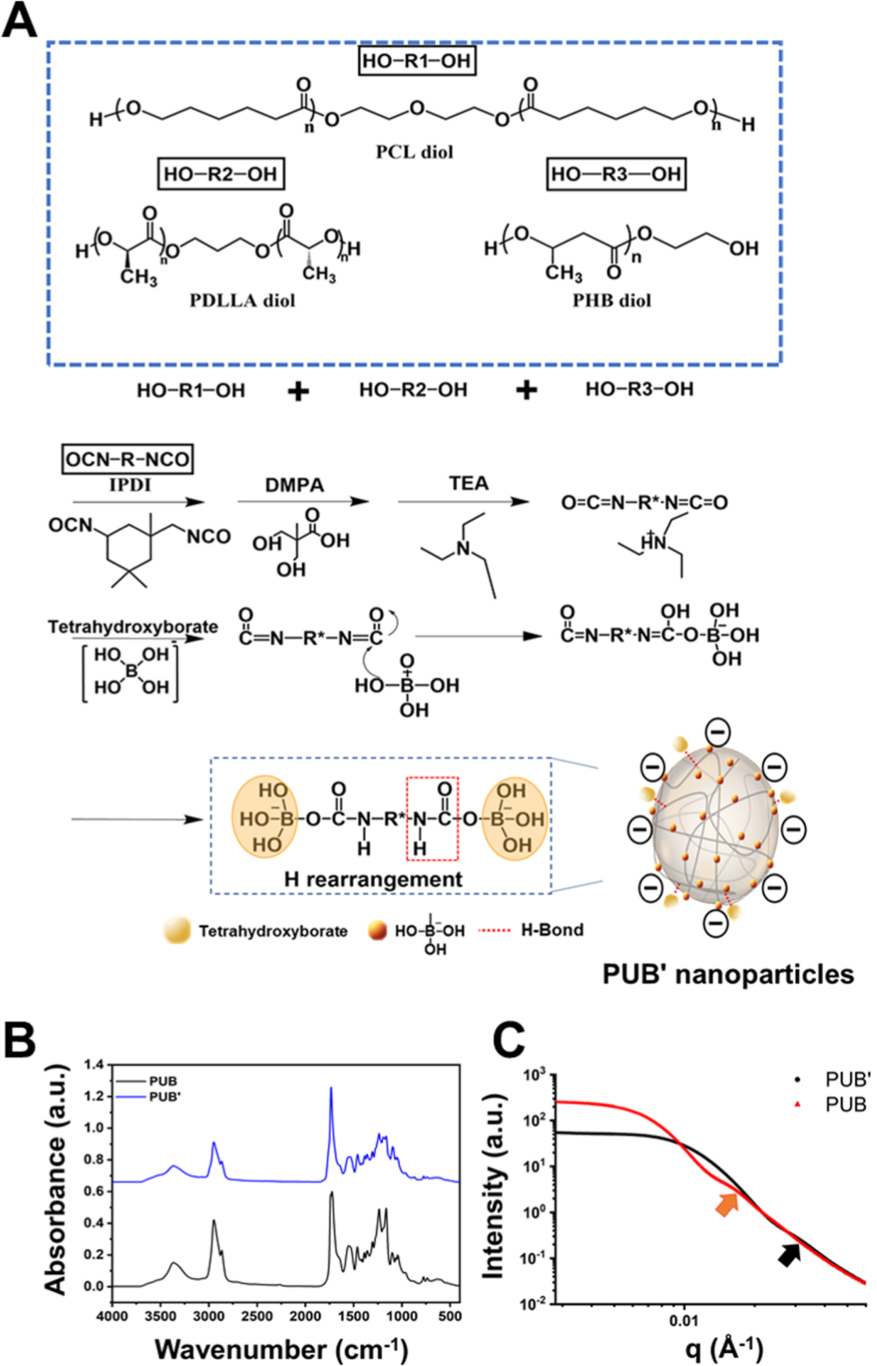
Synthesis of nanocross-linker PUB′. (A) Synthesis scheme for waterborne biodegradable PUB′. The difference between PUB and PUB′ was in the soft segment composition employed in the synthetic process, where the former was based on PCL oligodiol alone while the latter was based on a combination of 70 mol % PCL oliogodiol, 20 mol % PDLLA oliogodiol, and 10 mol % PHB oliogodiol. (B) FTIR spectra of PUB and PUB′. (C) Small-angle X-ray scattering (SAXS) profiles of PUB and PUB′ nanoparticles. The red arrow marks the first depression in the scattering curve for PUB nanoparticles, while the black arrow indicates the corresponding position for PUB′ nanoparticles.

The SAXS results for PUB and PUB′ dispersions are presented in Figure 2C. According to the SAXS analysis, the position of the first depression in the scattering curve of PUB′ (black arrow, *q* ∼ 3.0 × 10^–2^ Å^–1^) occurs at a higher *q* value than that of PUB (red arrow, *q* ∼ 1.5 × 10^–2^ Å^–1^). This shift suggests a smaller effective radius for the PUB′ nanoparticles in comparison to PUB nanoparticles, consistent with data from DLS. The Guinier analysis from SAXS experiments estimates the radius of gyration (*R*_g_) values of PUB and PUB′, as presented in Table 1. The *R*_g_ value of PUB (18.6 ± 1.5 nm) is greater than that of PUB′ (13.7 ± 1.6 nm). The shape factors (*R*_g_/*R*_h_) are also listed in Table 1. The shape factor for PUB is 0.96, indicating a structure close to a sphere (*R*_g_/*R*_h_ ∼ 0.77). In contrast, PUB′ has a shape factor of 1.10, suggesting a more elliptical form compared to PUB. The differences in *R*_g_ values and shape factors reflect the influence of soft segment composition on the structural characteristics of the nanoparticles. Specifically, the incorporation of PDLLA and PHB into the soft segments leads to variations in both size and morphology.

The parameters obtained from the optimal SAXS fitting of PUB and PUB′ nanoparticles are summarized in Table 2. The form factor revealed polar and equatorial radii (*R*_p_ and *R*_e_) of 27.6 and 26.1 nm for PUB, i.e., close to a sphere, and *R*_p_ and *R*_e_ of 19.5 and 16.9 nm for PUB′, i.e., ellipsoid. Besides, PUB nanoparticles were larger than PUB′ ones. The smaller correlation length (ξ) of PUB′ indicated a tighter and more compact internal structure compared to PUB. Transmission electron microscopy (TEM) images further corroborate the SAXS-derived shape contrast (Figure S1). Both PUB and PUB′ nanoparticles are well dispersed. PUB nanoparticles are predominantly quasi-spherical, with a mean size of ∼24.5 nm. By contrast, PUB′ particles are slightly elongated along one axis, giving an ellipsoidal morphology with a smaller mean size of ∼16.6 nm. These results confirmed the different mesoscale microstructures of PUB and PUB′ nanoparticles.

**Table 2:** Structural Parameters Derived from Model Fits to SAXS Curves for PUB and PUB′ Nanoparticles^a^

	form factor		axis ratio	correlation length
	*R*_p_ (nm)	*R*_e_ (nm)	*R*_p_/*R*_e_	ξ (nm)
PUB	27.6 ± 0.2	26.1 ± 0.2	1.05	4.5 ± 0.003
PUB′	19.5 ± 0.1	16.9 ± 0.1	1.15	3.6 ± 0.002

a *R*_p_: polar radius, *R*_e_: equatorial radius.

### 2.2 Preparation of PDUB and PDUB′ Hydrogels

The solutions of PEGDA and DTT were mixed to generate the PD precursor. The PUB or PUB′ dispersion as the nanocross-linker was then mixed with the PD precursor to prepare the PDUB or PDUB′ hydrogel through the formation of boronate ester bonds between PU–boronic acid and PD (Figure 3). The composition ratios of PDUB and PDUB′ hydrogels are listed in Tables 3 and S2. A previously reported glucose-sensitive hydrogel based on PD and pure tetrahydroxyborate (PDB hydrogel) and another hydrogel based on PD and tetrahydroxyborate with PEI (PDBI hydrogel)^14,15^ are also included in Table 3 for comparison. The gelation time of each PDUB and PDUB′ hydrogel was longer than that of PDB and PDBI hydrogels. Additionally, the gelation time increased as the tetrahydroxyborate content decreased. This result confirms that the inclusion of PUB and PUB′ effectively prolongs the gelation time, which may create a longer working window for 3D printing.

**Figure 3:**
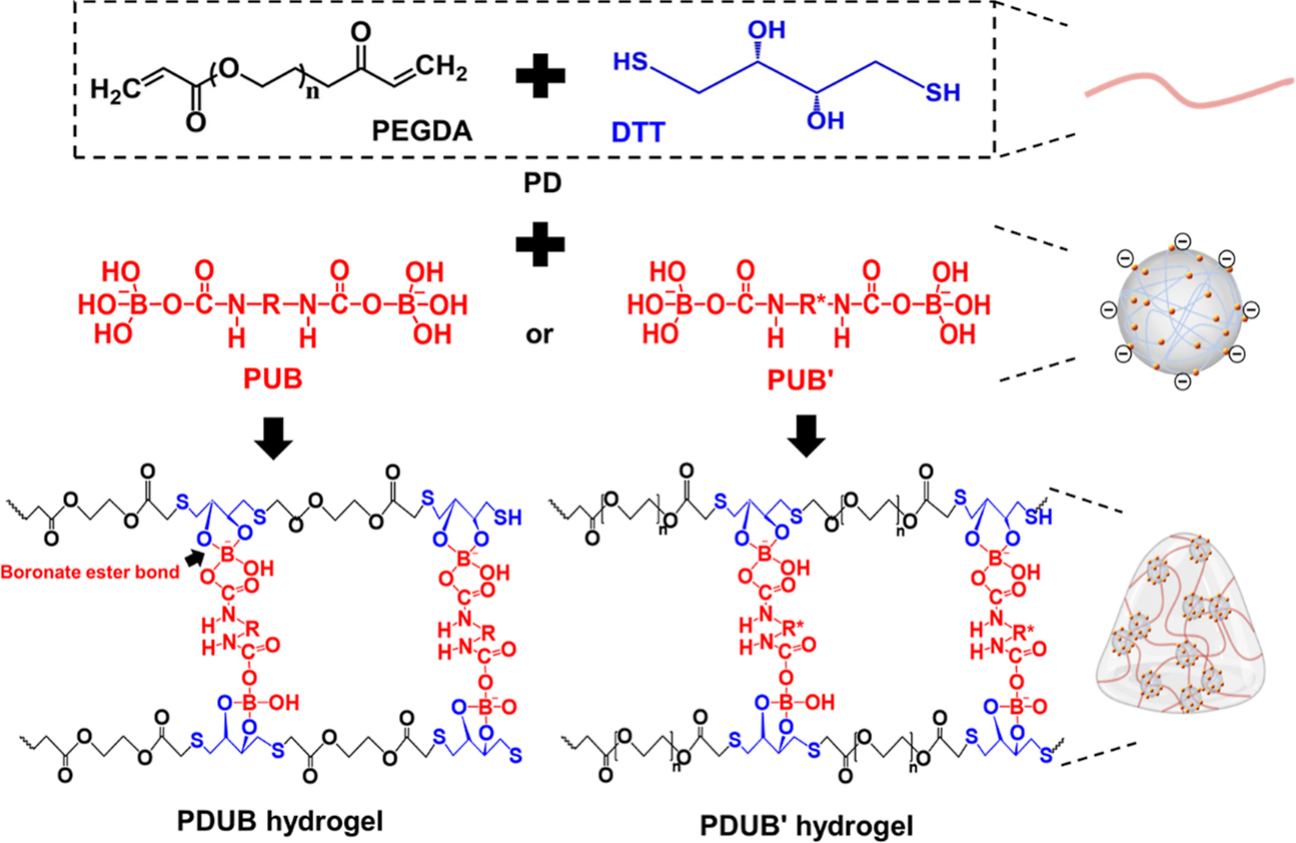
Preparation of PUB-based and PUB′-based hydrogels. PDUB and PDUB′ hydrogels were formed via boronate ester bonding. Initially, PEGDA was combined with DTT in the solution state, resulting in the formation of a PD precursor. Subsequently, the dispersion of PUB or PUB′ was blended with PD in different proportions to form PDUB or PDUB′ hydrogels.

**Table 3:** Composition and Gelation Time of PDUB and PDUB′ Hydrogels Prepared from PD and PUB (or PUB′)^a^

	PD (wt %)	PUB (or PUB′) (wt %)	borax contained in PUB (or PUB′) (wt %)	borax (wt %)	PEI (wt %)	gelation time (min)
PDB	12.2	0	0	1.9	0	∼1
PDBI	12.2	0	0	1.14	0.76	∼2.5
PDUB2	12.2	8.4	0.76	0	0	∼18
PDUB2′	12.2	8.4	0.76	0	0	∼13

a The control hydrogel (abbreviated as PDB in the table) was prepared from the same precursor (PD) with borax directly. Meanwhile, another hydrogel (abbreviated as PDBI in the table) prepared from PD and the borax/PEI mixture was included for comparison.

To clarify whether the observed gelation and printability arise from specific PU–borax interactions, we conducted control experiments using the mixtures of PD + PU and PD + borax + PU. The concentrations of borax and PU were matched to those of the PDUB2 and PDUB2′ hydrogel. PU used here refers to NH_2_-capped PU, distinct from the borax-containing PUB and PUB′ nanoparticles. The mixture of PD + PU failed to form a hydrogel, confirming that PU lacks cross-linking ability. When borax was added in the mixture of PD + PU, a brittle PD + borax + PU hydrogel formed rapidly (∼3 min), showing a gelation rate similar to that of the PD + borax hydrogel (∼2.5 min) (Table S3). Figure S2A,B shows the frequency sweep results for PD + borax and PD + borax + PU, respectively. Both hydrogels exhibited similar gel-like behavior, with the storage modulus (*G*′) consistently exceeding the loss modulus (*G*″) over the tested frequency range. However, steady shear viscosity measurements (Figure S2C,D) revealed that both groups displayed limited shear-thinning behavior. While the inclusion of PU slightly enhanced viscosity reduction under shear, it remained insufficient for extrusion through a 30G needle. These results indicate that, unlike the boronic acid-functionalized nanocross-linkers PUB and PUB′, the addition of NH_2_-capped PU does not effectively enhance the mechanical properties or injectability of the hydrogel. This confirms that PUB and PUB′ primarily participate in dynamic cross-linking through reversible boronate ester bonds, rather than acting merely as inert fillers.

The self-healing properties of PDUB hydrogels were assessed through visual observation, as shown in Figure S3A. The healing process of the hole was monitored over time after creating a 0.5 cm circular hole in the center of each hydrogel. The size of the holes in each hydrogel gradually decreased over time and fully self-healed to their original state within 60 min. The quantified self-healing rates based on changes in the hole diameter are presented in Figure S3B. The PDUB1 hydrogel showed a significant reduction in diameter within 5 min, while the other three hydrogels exhibited similar self-healing behavior at the 5 min mark. PDUB1 achieved complete healing within 10 min. PDUB2 showed significant hole healing in 20 min and was fully healed within 30 min. In contrast, PDUB3 and PDUB4 showed noticeable differences in the hole size after 30 min, and both achieved complete healing before 60 min.

### 2.3 Rheological Properties of PDUB and PDUB′ Hydrogels

The rheological properties of various PDUB hydrogels are presented in Figures 4 and S4. The *G*′ values of PDUB1, PDUB2, PDUB3, and PDUB4 at 25 °C were approximately 17.9, 4.28, 1.08, and 0.25 kPa, respectively, while the corresponding tan δ values were ∼0.32, ∼0.48, ∼0.58, and ∼0.68 (Figures 4A and S4A). The strain-dependent viscoelasticity experiments showed that PDUB2 (310%), PDUB3 (350%), and PDUB4 (530%) could endure strains higher than those of PDUB1 (130%), as shown in Figures 4B and S4B. Furthermore, all PDUB hydrogels except PDUB1 exhibited strain-hardening behavior. Damage-healing cycles were measured by applying strains exceeding the sol–gel transition point of each hydrogel. The *G*″ values of PDUB2, PDUB3, and PDUB4 hydrogels continued to decrease under prolonged strain, which indicates their thixotropic behavior. Meanwhile, all PDUB hydrogels after damage demonstrated approximately 100% self-healing efficiency within 500 s (Figures 4C and S4C). In the static shear experiments (Figures 4D and S4D), the steady shear viscosities of all PDUB hydrogels decreased as the shear rate increased. This observation confirms the shear-thinning behavior of PDUB hydrogels.

**Figure 4:**
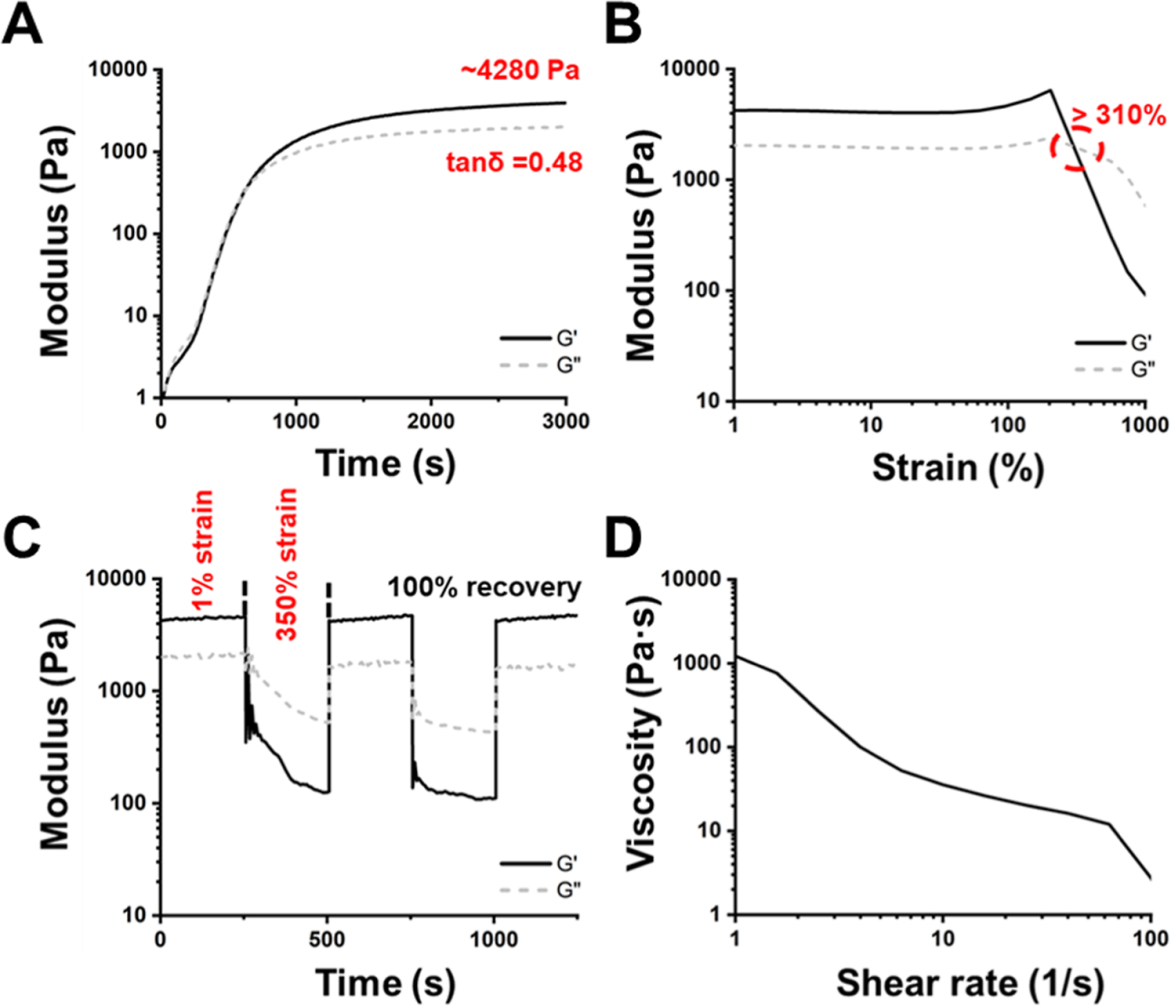
Rheological properties of the PDUB2 hydrogel. (A) Time-sweep experiments illustrating the storage modulus (*G*′) and loss modulus (*G*″) of the PDUB2 hydrogel over the gelling time, measured at 1 Hz and 1% strain during gelation at 25 °C. (B) Strain sweep (1–1000%) experiment evaluating strain-induced deconstruction at 25 °C and 1 Hz, with gel-to-sol transition occurring at strains ≥310%. (C) Damage-healing properties of the PDUB2 hydrogel demonstrated through two cycles of strain variation (1% → 350% → 1% → 350% → 1% strain) at 25 °C and 1 Hz. (D) Shear-thinning properties of the PDUB2 hydrogel determined by measuring the viscosity versus shear rate from 1 to 100 s^–1^ in a static (steady shear) experiment.

Through systematic composition optimization, the self-healing properties, mechanical performance, and rheological properties were fine-tuned to identify the optimal hydrogel for 3D printing. PDUB1 and PDUB2 hydrogels exhibited greater *G*′ values compared to PDUB3 and PDUB4 hydrogels, indicating better mechanical stability. This stability may benefit printing by ensuring structural integrity and reducing the risk of deformation. However, the rapid self-healing rate of the PDUB1 hydrogel may cause structural collapse during printing, making it unsuitable for high-resolution printing applications. Regarding toughness, PDUB2, PDUB3, and PDUB4 hydrogels demonstrated good stretchability, enduring higher strains before damage. Notably, PDUB2 achieved a suitable tan δ value, ensuring smooth extrusion through a needle while retaining structural integrity. Overall, the PDUB2 hydrogel emerged as the optimal formulation among PDUB hydrogels for 3D printing, considering mechanical strength, viscoelastic resilience, stretchability, self-repairing capabilities, and shear-thinning behavior. The PDUB2′ hydrogel was prepared by replacing PUB with PUB′ using the same composition ratio as the PDUB2 hydrogel for comparison and further optimization.

The rheological properties of PDUB2′ hydrogels are shown in Figure 5. The PDUB2′ hydrogel demonstrated a greater *G*′ of ∼6.45 kPa compared to the PDUB2 hydrogel (Figure 5A). During the frequency sweep, the *G*′ value of both PDUB2 and PDUB2′ hydrogels gradually stabilized as the frequency increased above 1 Hz while the *G*″ value declined (Figure S5), suggesting that the dynamic bonding of the network via the rather bulky nanocross-linker may be affected by the higher deformation frequency. Both hydrogels remained gel-like (*G*′ > *G*″) at all frequencies. For the PDUB2′ hydrogel, there was no evident strain-hardening and the critical damage strain was much lower (∼40% strain) than the PDUB2 hydrogel during the strain sweep (Figure 5B). Moreover, the self-healing efficiency of the PDUB2′ hydrogel was about ∼88% after damage for 500 s (Figure 5C), suggesting that dynamic bonding in the PDUB2′ hydrogel may be less effective compared to that in the PDUB2 hydrogel. Nevertheless, the PDUB2′ hydrogel may be suitable for 3D printing because of the appreciable tan δ value (∼0.51) comparable to PDUB2. The greater modulus and lower self-healing efficiency of PDUB2′ may also facilitate stackability and prevent structure collapse during 3D printing.

**Figure 5:**
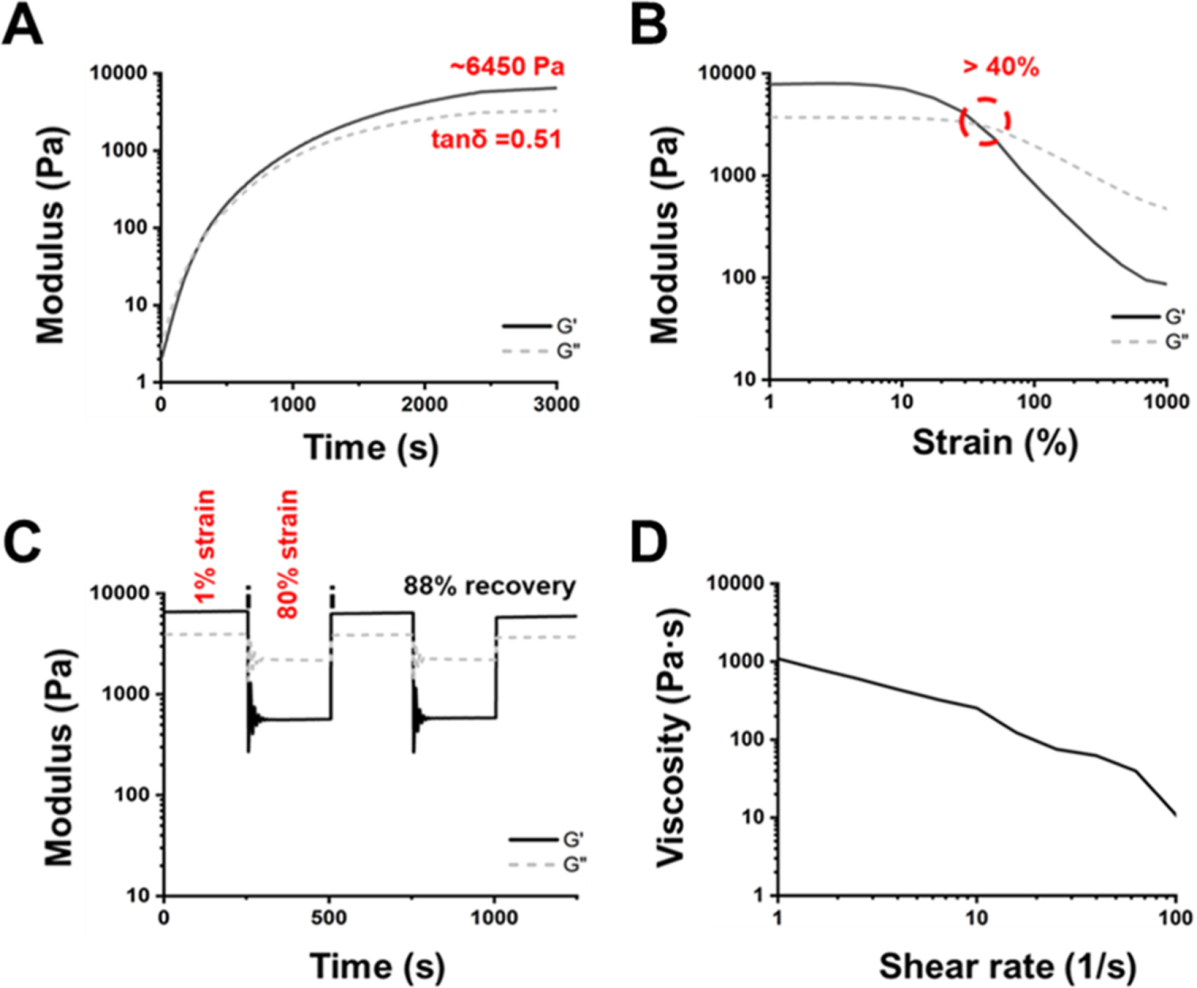
Rheological properties of the PDUB2′ hydrogel. (A) Time-sweep experiments illustrating the storage modulus (*G*′) and loss modulus (*G*″) of the PDUB2′ hydrogel over the gelling time, measured at 1 Hz and 1% strain during gelation at 25 °C. (B) Strain sweep (1–1000%) experiment evaluating strain-induced deconstruction at 25 °C and 1 Hz, with gel-to-sol transition occurring at strains ≥40%. (C) Damage-healing properties of the PDUB2 hydrogel demonstrated through two cycles of strain variation (1% → 80% → 1% → 80% → 1% strain) at 25 °C and 1 Hz. (D) Shear-thinning properties of the PDUB2′ hydrogel determined by measuring the viscosity versus shear rate from 1 to 100 s^–1^ in a static (steady shear) experiment.

To further quantify the shear-induced structural recovery of PDUB2 and PDUB2′ hydrogels, thixotropic viscosity recovery tests were performed using a three-interval time sweep protocol (Figure S6). Both hydrogels exhibited reversible shear-thinning behavior with a rapid viscosity decrease under high shear (60 s^–1^) and recovery upon reducing the shear rate to 1 s^–1^. Notably, PDUB2′ fully recovered its initial viscosity within approximately 200 s, whereas PDUB2 required nearly 300 s. This faster recovery suggests that PDUB2′ undergoes more efficient network reconstruction after shear disruption, which may enhance filament resolution and structural integrity during extrusion-based printing. The observed thixotropic behavior reflects dynamic network reformation mediated by reversible boronate ester bonds. This result is consistent with the modulus recovery observed in cyclic strain tests (Figures 4C and 5C), further confirming that the hydrogels possess intrinsic self-healing properties enabled by dynamic covalent cross-linking.

### 2.4 Time-Resolved Structural Changes during PDUB2 and PDUB2′ Gel Formation by Small-Angle X-ray Scattering and Transmission Electron Microscopy

SAXS was employed to analyze and contrast the real-time nanostructure changes of PDUB2 and PDUB2′ during the gelation process. The resulting scattering profiles and curves are listed in Figure 6. The mixture of linear precursor (PD) and nanocross-linker (PUB or PUB′) solutions was loaded into the instrument at time *t* = 0. As shown in Figure 6A,B, the scattering profiles of both PDUB2 and PDUB2′ hydrogels at *t* = 0 exhibited a distinct form factor (*q* ranging from ∼1.6 × 10^–2^ Å^–1^ to 1.8 × 10^–2^ Å^–1^). As the gelation process progressed, the form factors of both hydrogels decreased to varying degrees.

**Figure 6:**
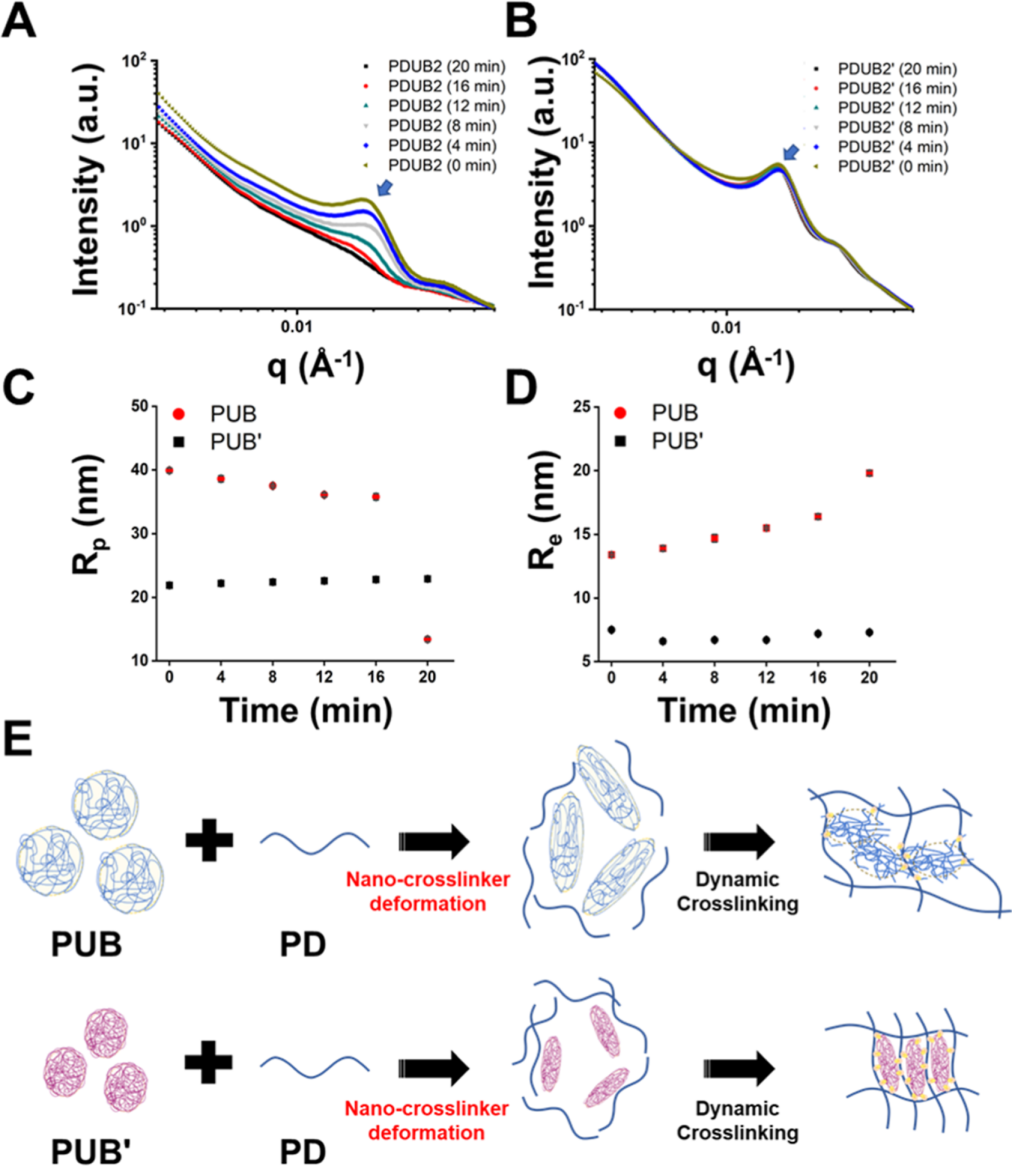
Time-resolved SAXS profiles recorded at 25 °C during gelation of the hydrogel. The SAXS profiles were collected at 4 min intervals for a period of 20 min following the mixing of PD precursor and PUB (or PUB′) nanoparticles to form the (A) PDUB hydrogel and (B) PDUB′ hydrogel. The blue arrow in (A,B) indicates the position of the first depression in the scattering curve, used to calculate the effective radius of the PUB and PUB′ nanoparticles (nanocross-linker). (C) Changes of polar radius for PUB and PUB′ nanocross-linker during gelation. (D) Changes of equatorial radius for PUB and PUB′ nanocross-linkers during gelation. (E) Hypothetical diagram illustrating the shape deformation of PUB and PUB′ nanocross-linkers during the gelation process. The reduced deformation of PUB′ nanocross-linkers suggests higher structural stability, which may correlate with the enhanced creep resistance observed in the PDUB2′ hydrogel (Figure 7).

To better understand the structural evolution of the network, the scattering data collected during gelation were further fitted using the model [eq 1] incorporating a power law for large-scale structural variations, an ellipsoid form factor with a hard-sphere structure factor for nanocross-linker analysis, and the Ornstein–Zernike equation to describe the local chain distance (Figure S7). Parameters derived from the optimal fitting are summarized in Tables 4 and 5. The intensity decays in the lower *q* range from 2.8 × 10^–3^ Å^–1^ to 5 × 10^–3^ Å^–1^, which is related to the fractal dimension (*D*_f_). The *D*_f_ quantifies the self-similarity and complexity of a structure. During the gelation process, both PDUB2 and PDUB2′ hydrogels exhibit an increase in *D*_f_ from the initial to the final stages, reflecting the development of a more complex and tightly packed network structure. The PDUB2′ hydrogel achieves a higher *D*_f_ and reaches equilibrium faster than the PDUB2 hydrogel.

**Table 4:** Structural Parameters Obtained from Model Fits to the SAXS Curves of the PDUB2 Hydrogel during Gelation^a^ ^,^ ^b^

time (min)	form factor		axis ratio	structure factor		power law	correlation length
	*R*_p_ (nm)	*R*_e_ (nm)	*R*_p_/*R*_e_	*R*_HS_ (nm)	η (%)	*D*_f_	ξ (nm)
0	39.9 ± 0.4	13.4 ± 0.1	2.98	15.4 ± 0.1	33 ± 0.04	2.98 ± 0.0008	2.1 ± 0.1
4	38.6 ± 0.5	13.9 ± 0.2	2.78	15.1 ± 0.1	33 ± 0.05	3.09 ± 0.0003	1.9 ± 0.1
8	37.5 ± 0.2	14.7 ± 0.3	2.55	14.9 ± 0.1	33 ± 0.01	3.15 ± 0.0003	1.7 ± 0.1
12	36.1 ± 0.3	15.5 ± 0.1	2.33	14.7 ± 0.2	29 ± 0.02	3.20 ± 0.0004	1.6 ± 0.1
16	35.8 ± 0.5	16.4 ± 0.2	2.18	14.5 ± 0.1	25 ± 0.03	3.20 ± 0.0001	1.6 ± 0.1
20	13.2 ± 0.4	19.8 ± 0.2	0.67	10.3 ± 0.2	18 ± 0.07	3.20 ± 0.0002	1.6 ± 0.1

a At *t* = 0, the mixture of precursor and nano-crosslinker solutions [PD and PUB] was loaded into the instrument.

b *R*_p_: polar radius, *R*_e_: equatorial radius, *R*_HS_: hard-sphere radius, η: volume fraction, *D*_f_: fractal dimension.

**Table 5:** Structural Parameters Obtained from Model Fits to the SAXS Curves of the PDUB2′ Hydrogel during Gelation^a^ ^,^ ^b^

time (min)	form factor		axis ratio	structure factor		power law	correlation length
	*R*_p_ (nm)	*R*_e_ (nm)	*R*_p_/*R*_e_	*R*_HS_ (nm)	η (%)	*D*_f_	ξ (nm)
0	21.9 ± 0.1	7.5 ± 0.1	2.92	18.8 ± 0.1	36 ± 0.05	2.92 ± 0.0001	2.7 ± 0.1
4	22.2 ± 0.1	6.6 ± 0.2	3.36	18.8 ± 0.2	37 ± 0.01	3.25 ± 0.0003	2.7 ± 0.2
8	22.4 ± 0.1	6.7 ± 0.1	3.34	19.0 ± 0.1	37 ± 0.04	3.25 ± 0.0003	2.7 ± 0.1
12	22.6 ± 0.2	6.9 ± 0.1	3.28	19.3 ± 0.1	37 ± 0.05	3.25 ± 0.0004	2.8 ± 0.1
16	22.8 ± 0.2	7.2 ± 0.1	3.17	19.6 ± 0.1	38 ± 0.07	3.25 ± 0.0003	2.9 ± 0.1
20	22.9 ± 0.2	7.3 ± 0.1	3.14	19.7 ± 0.1	38 ± 0.07	3.25 ± 0.0002	2.9 ± 0.1

a At *t* = 0, the mixture of precursor and nano-cross linker solutions [PD and PUB′] was loaded into the instrument.

b *R*_p_: polar radius, *R*_e_: equatorial radius, *R*_HS_: hard-sphere radius, η: volume fraction, *D*_f_: fractal dimension.

The observed hump in the *q* range from ∼1.5 × 10^–2^ Å^–1^ to 2.0 × 10^–2^ Å^–1^ (blue arrow) reflects the aggregation of PUB or PUB′ nanoparticles. The evolution of polar radius (*R*_p_) and equatorial radius (*R*_e_) over time for PUB and PUB′ nano cross-linkers are presented in Figure 6C,D, respectively. For the PUB nano cross-linker in PDUB2 hydrogels, *R*_p_ decreased from 39.9 ± 0.4 to 13.2 ± 0.4, while *R*_e_ increased from 13.4 ± 0.1 to 19.8 ± 0.2. The corresponding axis ratio reached a maximum of 2.98 at the initial stage and then decreased to 0.67 over time. In contrast, the PUB′ nanocross-linker in PDUB2′ hydrogels showed a modest increase in *R*_p_ from 21.9 ± 0.1 to 22.9 ± 0.2, accompanied by a slight decrease in *R*_e_ from 7.5 ± 0.1 to 7.3 ± 0.1. The axis ratio of the PUB′ nanocross-linker started at an initial value of 2.92, increased to a peak of 3.36 at 4 min, and then slightly decreased to 3.14. These observations indicate that both nanocross-linkers elongate initially, but subsequently the PUB′ nanocross-linker undergoes less morphological changes than the PUB nanocross-linker during the cross-linking and gelation process.

Changes in hard-sphere radius (*R*_HS_) and correlated volume fraction (η) of nanocross-linkers also play a vital role in defining the final gel structure. In PDUB2 hydrogels, the *R*_HS_ and η of PUB nanocross-linker decreased significantly (*R*_HS_ from 15.4 ± 0.1 nm to 10.3 ± 0.2 nm and η from 33% to 18%) during gelation. These changes reduce the effective packing density of the hydrogel network of PDUB2, leading to a less interconnected and structurally weaker gel matrix. Conversely, the PDUB2′ hydrogel exhibited a slight increase in both *R*_HS_ and η (*R*_HS_ from 18.8 ± 0.1 nm to 19.7 ± 0.1 nm and η from 36% to 38%), which indicated that the PUB′ nanocross-linker remained more structurally stable, with a denser packing of the nanoparticles. The compact arrangement is associated with the more interconnected and tighter hydrogel network of PDUB2′. The average local chain-to-chain distance (correlation length, ξ) in PUB and PUB′ nanocross-linkers was calculated using the Ornstein–Zernike equation [eq 8]. The ξ value of the PUB nanocross-linker decreased during gelation, while that of the PUB′ nanocross-linker did not change significantly.

Figure 6E illustrates the shape deformation process of PUB and PUB′ nanocross-linkers during gelation based on SAXS. Before mixing with the PD precursor, PUB nanoparticles exhibit a geometry close to spherical, while PUB′ nanoparticles display a slightly less isotropic shape. During gelation, the PUB nano cross-linker undergoes notable deformation (elongation) and gradually flattens down into a loosely organized form in the final stage. In contrast, the PUB′ nanocross-linker elongates and maintains its anisotropic configuration with minimal shape change afterward, which reflects the stronger resistance of the resulting hydrogel to deformation. These structural differences between PDUB2 and PDUB2′ hydrogels correspond to their distinct mechanical properties, as shown in subsequent analysis. In Figure S8, TEM images of the PDUB2 and PDUB2′ hydrogels support the SAXS-derived deformation scenario. In the PDUB2 hydrogel (Figure S8A), the PUB nanocross-linkers lose their spherical shape and flatten into loosely packed clusters, indicating the collapse of the original geometry. Conversely, the PDUB2′ hydrogel (Figure S8B) shows closely packed, individual PUB′ ellipsoids with little deformation, consistent with the higher particle volume fraction deduced from SAXS. This direct visualization further demonstrates the greater deformation resistance of the PUB′ nanoparticles and substantiates the mechanical performance of the PDUB2′ hydrogel.

Taken together, SAXS analyses reveal that PUB and PUB′ nano cross-linkers exhibit distinct shape and packing behavior during cross-linking, which strongly influence the resulting hydrogel network. These findings underline the critical role of morphology and packing behavior of the soft nano cross-linker in determining the mechanical stability and functional properties of the resulted hydrogel.

### 2.5 Self-Healing Rate and Time-Dependent Creep Behavior of PDUB and PDUB′ Hydrogels

The self-healing rates of PDUB2 and PDUB2′ hydrogels were compared through visual observation as shown in Figure 7A. According to the quantification in Figure 7B, the PDUB2 hydrogel demonstrated significant healing of the drilled hole within 20 min and achieved complete recovery within 30 min. In contrast, the PDUB2′ hydrogel required up to 60 min for complete healing. These results indicate that the PDUB2′ hydrogel exhibits lower self-healing efficiency than the PDUB2 hydrogel, consistent with the rheological data.

**Figure 7:**
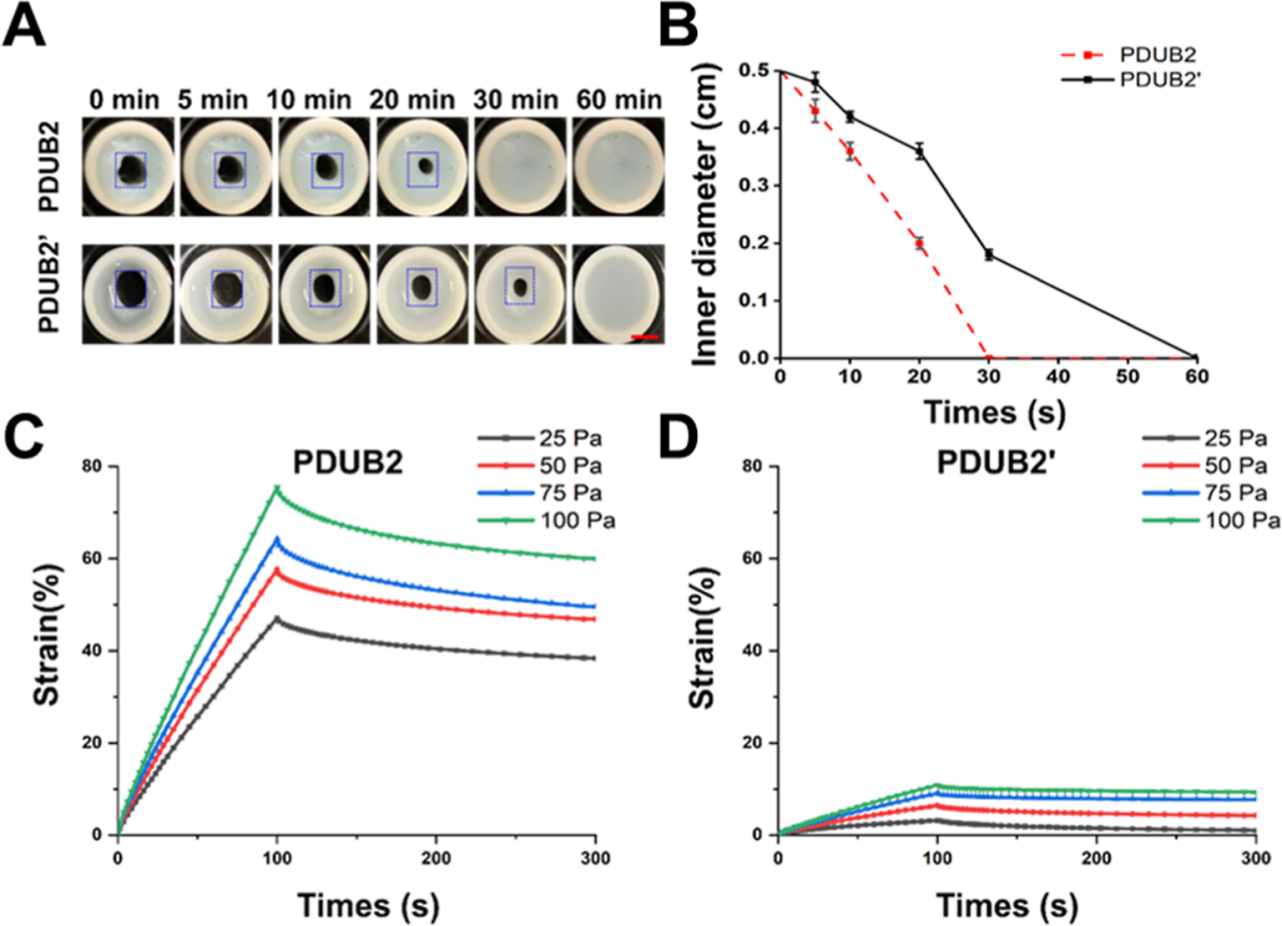
Self-healing and creep behavior of PDUB2 and PDUB2′ hydrogels. (A) Macroscopic comparison of self-healing rates between PDUB2 and PDUB2′ hydrogels. The highlighted squares indicate the location of the drilled holes. Scale bar, 0.5 cm. (B) Quantification of self-healing rates based on variation in hole diameter over time. (C) Creep and creep recovery (strain vs time) curves for the PDUB2 hydrogel under applied stresses of 25, 50, 75, and 100 Pa (within the linear viscoelasticity region) at 25 °C. (D) Creep and creep recovery curves for the PDUB2′ hydrogel under the same applied stress levels. Both hydrogels were tested at 25 °C, with each cycle including 100 s of creep followed by 200 s of recovery.

The PDUB2′ hydrogel (Figure 7C) showed significantly better creep resistance (lower creep strain) and more strain recovery than the PDUB2 hydrogel under varying applied stresses (Figure 7D). The creep resistance and recovery behavior of PDUB2′ also allow the hydrogel to maintain structural integrity under stress. Meanwhile, the PDBI hydrogel developed in prior literature exhibited intermediate creep behavior in-between PDUB2 and PDUB2′ hydrogels (Figure S9). Upon stress removal, the PDBI hydrogel demonstrated an immediate strain recovery, i.e., a sudden drop in the strain, due to its quick self-healing behavior. This recovery behavior indicates reversible deformation within the network, which could affect its ability to fix the shape during 3D printing.

### 2.6 Glucose Sensitivity and Cytocompatibility

PUB and PUB′ nanoparticles, as well as their corresponding hydrogels PDUB2 and PDUB2′, were stained with VB-48/PI to evaluate the viability of encapsulated endothelial cells (ECs) (Figure 8A–D). For the nanoparticles, PUB exhibited 88.1% healthy cells and 7.7% dead cells, while PUB′ had slightly better results with 90.4% healthy cells and 5.7% dead cells. In the hydrogel groups, the PDUB2 hydrogel had good cytocompatibility, with 91.7% healthy cells and 4.9% dead cells. The PDUB2′ hydrogel showed the highest cytocompatibility, which achieved 92.6% healthy cells and only 4.3% dead cells. The control PDB and PDBI hydrogels showed higher cytotoxicity, with PDBI exhibiting slightly better cytocompatibility than PDB (Figure S10). These results demonstrate that incorporating a long-chain soft nanocross-linker significantly improves the cytocompatibility of the hydrogels. Among the tested samples, the PDUB2′ hydrogel displayed the most favorable EC viability, with the highest percentage of healthy cells and the lowest percentage of dead cells.

**Figure 8:**
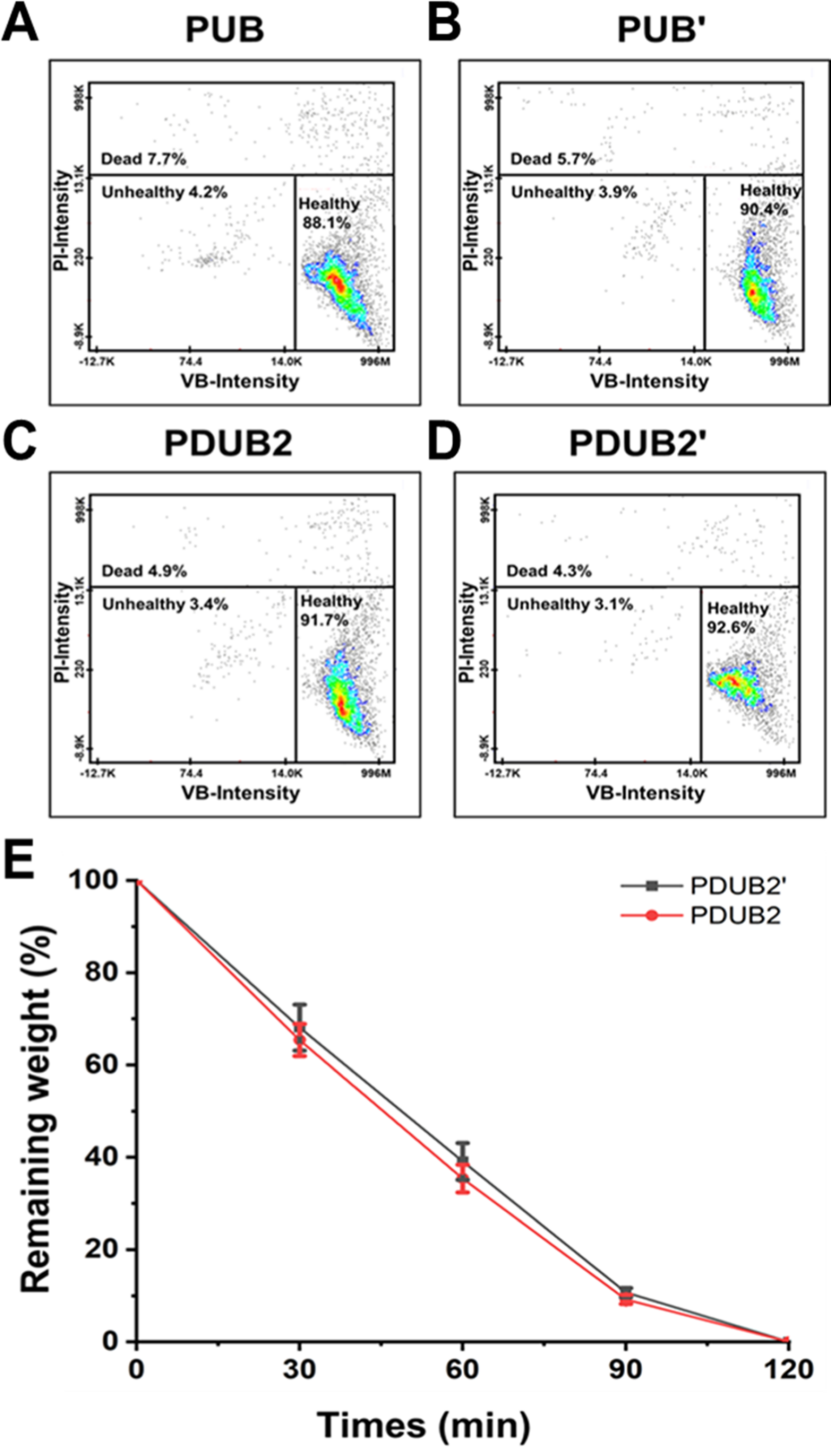
Cytocompatibility and glucose sensitivity of PDUB2 and PDUB2′ hydrogels. (A–D) Cytocompatibility analyses for the nanocross-linker (PUB and PUB′ nanoparticles) as well as their corresponding hydrogels (PDUB2 and PDUB2′ hydrogels) using VB-48 staining to evaluate the viability of the laden ECs. (E) The remaining weight of PDUB2 and PDUB2′ hydrogels plotted against dissolution time during glucose-induced sacrificial dissolution. Both hydrogels exhibited similar time-dependent dissolution trends, with complete dissolution within 120 min.

The glucose sensitivity of PDUB2 and PDUB2′ hydrogels was evaluated by monitoring the remaining weight during the sacrificial dissolution under identical total solid content conditions (Figure 8E). Both hydrogels exhibited a time-dependent dissolution, with the remaining weight gradually decreasing over 120 min. The similar sacrificial behavior of PDUB2 and PDUB2′ hydrogels suggests that the total solid content may be the dominant factor influencing the dissolution time. According to literature,^15^ the cytocompatible hydrogel that sacrifices in 120 min is suitable to carry cells, be eluted for cell attachment to a nonsacrificial hydrogel, and produce vascular-like channels within a nonsacrificial hydrogel.

### 2.7 Stackability and Printing Resolution of PDUB and PDUB′ Hydrogels

PDUB2 and PDUB2′ hydrogels were 3D printed using a 30G nozzle (inner diameter = 160 μm) on a platform at 25 °C, with a designed size of 1.2 cm (*L*) × 1.2 cm (*W*) × 1.2 cm (*H*). The side view of the printed PDUB2 and PDUB2′ hydrogels is presented in Figure 9A. The PDUB2 structure exhibited significant collapse at higher layers, with a final height of 0.4 cm and an outward expansion at the base. In contrast, the PDUB2 hydrogel maintained a stable final height of about 1 cm. The previously reported PDB hydrogel could not be printed through the 30G nozzle. While another previously reported PDBI hydrogel was printable through the 30G nozzle, the limited working window required printing in 3 min before complete gelation. Besides, the PDBI hydrogel was more suitable for gel-in-gel printing. The printed height of the PDBI hydrogel (if not gel-in-gel) would collapse to approximately 0.2 cm as shown in Figure S11.

**Figure 9:**
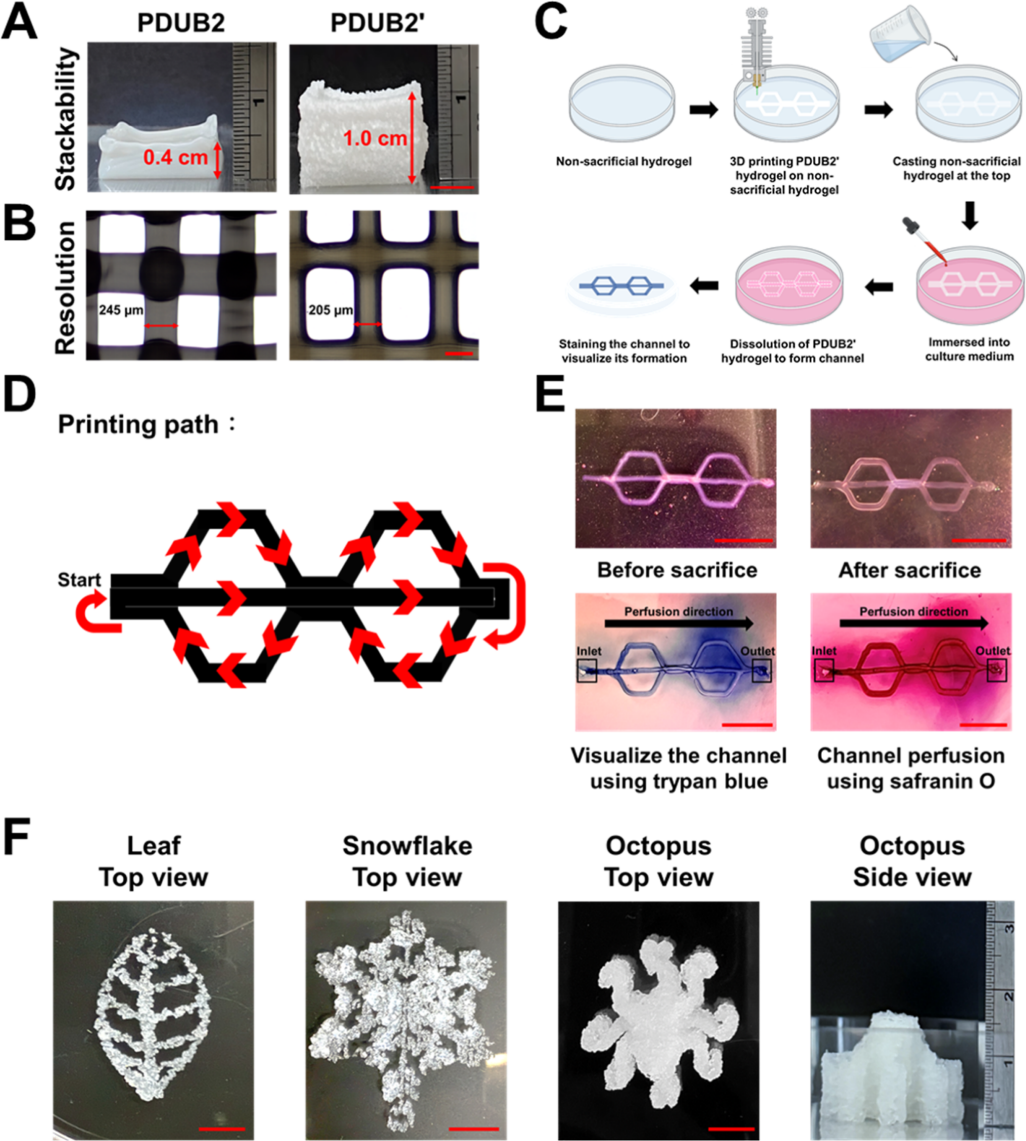
3D printability of PDUB2 and PDUB2′ hydrogels. (A) Side views of 3D-printed PDUB2 and PDUB2′ hydrogel constructs, using a 160 μm nozzle. Scale bar, 0.5 cm. The number of deposited layers was 60 for both constructs. (B) Microscopic images revealing the size of printed filaments for PDUB2 and PDUB2′ hydrogel constructs. Scale bar, 200 μm. (C) Schematic illustration of the channel formation process. (D) The printing path showing the predefined trajectory for PDUB2′ hydrogel deposition. The pattern was printed for three layers. (E) Formation of perfusable channels, illustrating the removal of the PDUB2′ hydrogel by culture medium to generate hollow structures. Trypan blue solution was first perfused to dye the channel architecture, while safranin O solution was perfused as a second dye to replace the first blue dye and confirm the channel patency. Scale bar, 0.5 cm. The hollow channels in (E) were fabricated using a 260 μm nozzle and deposited for three layers. The color near the outlet was due to the dye accumulation at the exit of the channel. (F) 3D printing of geometrically complex constructs using the PDUB2′ hydrogel. These complex architectures illustrate the capacity of the hydrogel to preserve shape fidelity and support its own weight postprinting. These constructs were printed using a 260 μm nozzle. Scale bar, 1 cm. The number of deposited layers was 60 for the octopus construct.

Microscopic imaging (Figure 9B) revealed that the lateral size of the extruded PDUB2′ hydrogel was approximately 205 μm (extrusion expansion ratio ∼28%), which is closer to the inner diameter of the 30G needle. This indicates that the PDUB2′ hydrogel has a lower extrudate swelling than the PDUB2 hydrogel, which showed a lateral size of approximately 245 μm (extrusion expansion ratio ∼53%).

Figure 9C illustrates the channel formation process using PDUB2′ as the sacrificial hydrogel. Initially, a nonsacrificial gelatin hydrogel was prepared as the base, onto which the PDUB2′ hydrogel was 3D printed and stacked for 3 layers. After printing, the printed structure was embedded in gelatin by placing more gelatin on the top. The embedded structure was then immersed in culture medium, which facilitated the dissolution of the PDUB2′ hydrogel to form the channel. The channel was then stained with a trypan blue solution infusion for visualization. Figure 9D illustrates the printing path of PDUB2′ hydrogel, where the predefined deposition trajectory ensured precise filament alignment and promoted the formation of an interconnected structure. Figure 9E further evaluates the formation of the patent channels. Following the elution of the PDUB2′ hydrogel, well-defined hollow channels were successfully obtained while maintaining the intended structure. Trypan blue staining clearly delineated the channel architecture. Safranin O was subsequently infused as a second stain to confirm that the channels remained open, as indicated by the uniform replacement of trypan blue by safranin O dye throughout the structure of the channels.

To further evaluate the printability and shape retention capability of PDUB2′ hydrogels, a series of complex 3D structures—including a leaf, a snowflake, and an octopus—were successfully printed (Figure 9F). These constructs maintained their predefined geometries, demonstrating the ability of the hydrogel to retain its structural fidelity after extrusion. Notably, the printed octopus structure achieved a vertical stacking height of approximately 1.8 cm without collapse, underscoring the capacity of the hydrogel for supporting multilayer structures.

## 3 Discussion

Although self-healing hydrogels are injectable and generally considered candidates for 3D printing inks, their self-healing property can result in low creep resistance. To maintain the shape after printing, postprinting secondary cross-linking is often necessary.^30,31^ A printable self-healing hydrogel with creep resistance and stackability may be developed using the concept of nanocomposites, where nanoparticles, particularly rigid inorganic nanoparticles such as silicates and metallic nanoparticles, can be used to enhance the creep resistance.^28,32^ These nanocomposite systems primarily rely on the high packing density and rigidity of inorganic particles to reinforce the network.^32,33^ Research studies on using soft, elastic nanocross-linkers to improve the 3D stacking are less documented so far. Meanwhile, organic nanoparticles based on boronic acid-based dynamic click chemistry have been developed for drug delivery,^34,35^ but they have not been designed to cross-link a gel network so far.

In the present study, two different boronic acid-functionalized PU nanoparticles, i.e., PUB and PUB′, were synthesized and their performances as nanocross-linkers compared. The soft segment in PUB consists solely of a PCL oligomer, while PUB′ incorporates a blend of PCL, PDLLA, and PHB oligomers in its soft segment. The PHB polymer is a microbially derived aliphatic polyester with a mechanical strength comparable to polypropylene.^36^ The regular stereochemical structure of PHB contributes to its high crystallinity,^37,38^ promoting phase separation between the soft and hard segments by enabling PHB in PUB′ to self-aggregate into ordered crystalline domains.^22^ This enhanced crystallinity may result in distinct structural conformation of PUB′ compared to PUB. Based on our results, the incorporation of PHB leads to a more compact arrangement of the polymer chains in PUB′, which may limit the infiltration of water molecules. The shape factor (*R*_g_/*R*_h_) is introduced to further characterize the particle morphology. This dimensionless value provides insights into the shape of nanoparticles.^39^ A higher shape factor typically indicates a more elongated particle structure with a greater directional alignment. The shape factor of homogeneous spherical nanoparticles is approximately 0.77, while rod-like particles have a shape factor around 1.5.^40,41^ The larger shape factor of PUB′ indicates that it is more elongated than the PUB morphology and closer to an ellipsoid. This elongated shape of PUB′ may facilitate the stacking of polymer chains along their major axis.^41^ Meanwhile, PUB tends to adopt a more spherical structure with a less chain orientation. These findings of morphological differences are supported by SAXS fitting results. Additionally, SAXS fitting results provide evidence for the difference of the internal microstructure. The results of ξ indicate that PUB′ has a more compact internal structure, likely due to the ordered alignment of polymer chains and the particle geometry. In contrast, PUB has a less organized internal structure with fewer interactions between polymer chains. This difference in microstructure could affect the performance of the nanoparticles in serving as cross-linkers.

Rheological properties play a critical role in evaluating the 3D printability of hydrogels.^42,43^ The rheological properties of PDUB2 and PDUB2′ hydrogels exhibited significant differences. The higher *G*′ of PDUB2′ hydrogels can be attributed to the unique characteristics of PUB′ as the nanocross-linker. PUB′ nanoparticles have a more compact internal structure, which increases the network density of the PDUB2′ hydrogel and thus the mechanical strength. Meanwhile, the PDUB2′ hydrogel has a tan δ value similar to that of PDUB2, indicating a balanced energy storage and dissipation capacity and suggesting the potential of both hydrogels for extrusion-based 3D printing. The self-healing behavior of the hydrogels in this study primarily originates from the reversible boronate ester bonds formed between the PUB or PUB′ nanocross-linkers and the PD polymer chains. The formation and dissociation of dynamic covalent bonds rely on sufficient chain mobility to facilitate effective functional group interactions and the self-healing properties of the materials.^44^ Upon mechanical damage, these dynamic cross-links can temporarily dissociate, but their rapid reformation capability allows the broken network to self-repair through chain diffusion and recross-linking. Self-healing depends on dynamic covalent boronate ester formation, in which boronic acid and diol groups undergo reversible condensation via a tetracoordinate boronate intermediate in aqueous conditions near physiological pH, allowing bonds to break upon damage and reform rapidly to restore network integrity.^45^ In the PDUB2 hydrogel, the PUB nanocross-linker contains soft segments with relatively high chain mobility, which facilitates the rapid reformation of dynamic boronate ester bonds. As a result, PDUB2 exhibits superior recovery of the mechanical modulus and more effective macroscopic healing. Meanwhile, the PUB′ nanocross-linker for the PDUB2′ hydrogel contains crystallizable PHB oligomers in its soft segments. The formation of crystalline domains in PDUB2′ restricts the chain mobility, thereby slowing down the recross-linking process and leading to reduced healing efficiency.

The morphological deformation of a soft nanocross-linker such as PUB and PUB′ during gelation plays a crucial role in determining their η and the mechanical performance of the resulting hydrogel. Time-resolved SAXS analysis reveals that PUB and PUB′ nanocross-linkers undergo distinct shape deformation processes during gelation, accounting for the structural differences observed between PDUB2 and PDUB2′ hydrogels. The PUB nanocross-linker undergoes significant morphological transformation, as evidenced by a decrease in *R*_p_ and an increase in *R*_e_. This deformation indicates that PUB nanoparticles keep changing the shape during the cross-linking reaction, including spatial redistribution and partial collapse that directly influence the network structure. Although such marked deformability may facilitate functional group rearrangement and enhance dynamic properties (e.g., self-healing), it may compromise the mechanical stability of the hydrogel under prolonged loading or multilayer stacking, as evidenced by the low creep resistance and recovery. In contrast, the PUB′ nanocross-linker undergoes relatively irreversible deformation during gelation and maintains the ellipsoidal morphology throughout the process, leading to a more than 2-fold higher volume fraction (η ∼ 38%, compared to ∼18% for PUB). This much denser packing directly facilitates the formation of a more compact and robust hydrogel network, as reflected by the higher *D*_f_ in SAXS observation. Such a tightly packed network enhances the mechanical performance of the PDUB2′ hydrogel, contributing to superior creep resistance and 3D stackability. Notably, the rise in the axis ratio of the PUB′ nanocross-linker from 0 to 4 min may correspond to the rapid and intense cross-linking reaction during the early stage of gelation. Meanwhile, the ξ of the PUB nanocross-linker decreases as gelation progresses, reflecting reduction of interchain spacing. The ξ change for the PUB′ nanocross-linker is less pronounced, further supporting the higher structure stability and creep resistance of the network. Time-resolved SAXS analysis shows that PUB′ nanocross-linkers have better ellipsoidal shape retention, higher η, and increased *D*_f_, which indicate more ordered and compact nanoparticle arrangement within the network. During gelation, this compact arrangement enhances the local density of dynamic boronate ester cross-links, leading to a denser and more robust hydrogel network. As a result, the PDUB2′ hydrogel exhibits a higher shear modulus (Figure 5A) and greater creep resistance (Figure 7D). These microstructural advantages also translate to enhanced extrusion-based 3D printing. The printed PDUB2′ filaments can better maintain their intended geometry and support subsequent layers (Figure 9A,B).

The ability to construct high-resolution filaments is important for 3D bioprinting, where structural integrity and mechanical stability directly impact the long-term functionality. As a sacrificial template to produce hollow microchannels in a construct, the PDUB2′ hydrogel exhibited superior geometric retention after printing, maintaining channel morphology throughout the sacrifice process. This behavior can be attributed to its stable network structure, which effectively prevented collapse and ensured postprinting shape fidelity. Extrusion expansion is another critical parameter for evaluating the printing performance of hydrogels. Excessive expansion can cause filament diameter deviations, compromising accuracy and stability.^46^ The reduced extrusion expansion as well as better stackability observed in the PDUB2′ hydrogel compared to the PDUB2 hydrogel may be attributed to the rigid network structure formed by the PUB′ nanocross-linker with twice hard-sphere packing density. The rigid network improves the cross-linking stability and restricts the mobility of local chain segments, reducing elastic recovery during extrusion. Compared to PDB and PDBI hydrogels reported in prior literature,^14,15^ the new PDUB2 and PDUB2′ hydrogels offer notable advantages. As a matter of fact, the PDB hydrogel is not extrudable by the 30G nozzle. The PDBI hydrogel is printable by the 23G nozzle but has a working window of about three min to be printed by the 30G nozzle, which restricts its practical applicability.

In summary, we successfully designed novel boronic acid-functionalized PU nanocross-linkers PUB and PUB′, highlighting the significant advantages of using long-chain soft nanocross-linkers in constructing the dynamic boronate ester hydrogel system. The incorporation of PHB oligomers in PUB′ endowed the nanocross-linker with enhanced crystallinity and a more compact internal structure, which led to superior morphological constancy and cross-linking efficiency of the hydrogel network. These properties significantly improved the extrusion stability, stacking consistency, creep resistance, and shape retention of the resulting hydrogel during 3D printing. Meanwhile, time-resolved SAXS provides critical insights into the morphological evolution and internal structure differences of PUB′ versus PUB throughout the cross-linking process and demonstrates how the internal structure contributes to cross-linking efficiency, morphological stability, packing density, and creep behavior of the network, which directly correlates with the enhanced hydrogel performance. Moreover, the introduction of long-chain soft boronate ester nanocross-linkers establishes a theoretical framework for producing low-toxicity glucose-sensitive hydrogels with potential applications in sacrificial materials, controlled drug carriers, and smart sensing technologies, offering a new pathway for designing soft nanocross-linkers for intelligent hydrogel systems.

## 4 Conclusions

New boronic acid-functionalized PU soft nanoparticles PUB and PUB′ were synthesized and successfully served as dynamic nanocross-linkers for PEGDA–DTT (PD) to form glucose-sensitive hydrogels (i.e., PDUB and PDUB′). SAXS highlighted distinct morphological differences between PUB and PUB′ before and during gelation. PUB exhibited a spherical morphology (shape factor, *R*_g_/*R*_h_ = 0.96) with significant shape deformation (axis ratio *R*_p_/*R*_e_ reduced from 2.98 to 0.67) during gelation, resulting in lower final packing density (∼18% volume fraction) within the network. In contrast, PUB′ displayed an ellipsoidal morphology (*R*_g_/*R*_h_ = 1.10) and maintained a stable shape during gelation (axis ratio *R*_p_/*R*_e_ slightly increased from 2.92 to 3.14), forming a more densely packed hydrogel network (∼38% volume fraction). The PDUB2 hydrogel had moderate shear modulus (*G*′ ≈ 4.28 kPa), fast self-healing (∼100% efficiency), but limited creep resistance under sustained load. The PDUB2′ hydrogel had greater modulus (*G*′ ≈ 6.45 kPa), superior creep resistance under 100 Pa shear stress, and slightly lower self-healing efficiency (∼88%), associated with the restricted chain mobility and compact internal structure of PUB′. Both hydrogels showed glucose sensitivity and EC viability (>90%). The 3D printability of PDUB2′ hydrogels significantly outperformed that of PDUB2 in terms of filament stackability and resolution. The printed PDUB2′ maintained a stacking height of over 1 cm with small extrusion expansion. Hollow channels with precise dimensions can be fabricated using PDUB2′, demonstrating its feasibility as an advanced sacrificial template for producing vascular-like structures. Overall, the PU-boronic acid nanocross-linker, particularly PUB′, provides glucose sensitivity and noncytotoxicity to the hydrogel network, showing morphological adaptability during the dynamic cross-linking process. The dynamic morphological evolution of the nanocross-linker revealed by SAXS and model fitting also provides insights and establishes new criteria for designing a 3D stackable composite bioink with high packing density (>36% volume fraction) using soft, deformable nanocross-linkers.

## 5 Materials and Methods

### 5.1 Synthesis of PUB and PUB′

The soft segment used in the PUB synthesis was PCL oligodiol (*M*_n_ 2000 Da, Sigma-Aldrich). In contrast to the previously developed biodegradable NH_2_-capped PU, the synthesis of PUB was performed by replacing one of the chain extenders EDA with borax (Sigma-Aldrich, USA). Initially, the PCL diol was introduced into a four-neck round vessel (250 mL) placed in an oil bath and stirred at 180 rpm under a nitrogen atmosphere at 75 °C for 30 min. Subsequently, the hard segment isophorone diisocyanate (IPDI, Evonik Degussa GmbH, USA) and tin­(II) 2-ethylhexanoate (T-9, Alfa Aesar) catalyst were added to carry out the prepolymerization for 3 h. After that, methyl ethyl ketone (MEK, J. T. Baker) as the solvent and 2,2-bis­(hydroxymethyl)­propionic acid (DMPA, Sigma, USA) as the chain extender were introduced into the reaction vessel and refluxed at 75 °C for 1 h followed by cooling to 50 °C. Triethylamine (TEA, J. T. Baker, USA) was added in an equimolar quantity to DMPA to neutralize the carboxyl groups of DMPA for 30 min. Finally, borax, which dissolved in deionized water to form tetrahydroxyborate, was added to the vessel under vigorous stirring (1186 rpm) for a uniform dispersion. The stoichiometric ratio of oliogodiol/IPDI/DMPA/borax based on normality was 1:3.52:1:0.76. The residual MEK and TEA in the mixture were removed through vacuum distillation. After the residual solvent and neutralizing agent were eliminated by vacuum distillation, the final water dispersion of PUB was obtained. The solid content was about 30% and was adjustable by adding deionized water. A second boronic acid-functionalized PU PUB′ was synthesized similarly to PUB, but at a higher polymerization temperature of 115 °C. Unlike PUB where the soft segment contains PCL oligodiol alone, PUB′ has a more sophisticated soft segment composition, which is a combination of 70 mol % PCL oligodiol, 20 mol % poly­(d,l-lactide) (PDLLA) oligodiol (*M*_n_ ∼ 1500 Da), and 10 mol % poly­(3-hydroxybutyrate) (PHB) oligodiol (*M*_n_ ∼ 1500 Da). PDLLA and PHB oligodiols were obtained by synthesis. PDLLA oligodiols were synthesized through the ring-opening polymerization of lactide (Purace) with propane-1,3-diol (Alfa Aesar, UK), using tin­(II) 2-ethylhexanoate (Sn­(Oct)_2_) as a catalyst. The PHB oligomer was synthesized using (Ningbo TianAn Biological Material, China) and purified by recrystallization. The purified oligomer was then dissolved in 1-methoxy-2-(2-methoxyethoxy)­ethane under a nitrogen atmosphere at 155 °C. Subsequently, it reacted with dibutyltin dilaurate (Alfa Aesar, USA) and ethane-1,2-diol for a duration of 4 h. After the reaction, the oligomer underwent a series of refinement steps, including abstraction, precipitation, and filtration.^22^ The three kinds of oligodiols were put together and stirred under a nitrogen atmosphere at 115 °C for 30 min to ensure uniform mixing, followed by the later synthetic procedures. All PUB and PUB′ nanoparticles in this study were dialyzed (MWCO 12–14 kDa) for three days after synthesis to remove unreacted borax and low-molecular-weight impurities prior to hydrogel formulation.

The hydrodynamic radius (*R*_h_), PDI, and zeta potential values of PUB and PUB′ dispersions in water were measured at 25 °C by a particle analyzer (Delsat Nano, Beckman Coulter) equipped with DLS and electrophoretic light scattering. The molecular weights of PUB and PUB′ were assessed through a GPC (JASCO, Japan) system equipped with a refractive index detector (RI-930). The PUB and PUB′ dispersions were characterized by FTIR (Perkin-Elmer, USA). The FTIR analysis was conducted over the wavenumber range of 800–4000 cm^–1^.

### 5.2 Preparation of Glucose-Sensitive Hydrogels Based on PUB and PUB′

Polyethylene glycol diacrylate (PEGDA, *M*_n_ = 700 gmol^–1^) and the solid powder of DTT were purchased from Sigma-Aldrich (USA, Canada respectively). PEGDA (20 wt %) and DTT (4 wt %) were dissolved in 0.5 mL of deionized water under vigorous stirring for 1 h. DTT could undergo a thiol–ene Michael addition reaction with PEGDA, which resulted in the formation of a PEGDA–DTT polymer (abbreviated PD in this study). Subsequently PD and PUB (or PUB′) were mixed in different ratios to obtain the PUB (or PUB′)-based hydrogel (abbreviated as PDUB or PDUB′ hydrogel). In the hydrogel, PD is the linear precursor and PUB (or PUB′) serves as a nanocross-linker for the network. On the other hand, the glucose-sensitive hydrogel based on pure tetrahydroxyborate (PDB) and that based on tetrahydroxyborate with PEI (PDBI) previously reported in literature^15^ were selected as the control group and the comparison group for the current study.

### 5.3 Rheological and Creep Measurement

The rheological properties of hydrogels were measured by a dynamic rheometer (HR-2, TA Instruments) with a 2° angle cone (40 mm diameter) geometry at 25 °C. Two distinct modes of rheological tests were conducted: dynamic shear (time-sweep) and static shear experiments. *G*′ and *G*″ of the time-dependent measurement were measured at 1% oscillating shear strain and 1 Hz frequency. In the frequency sweep, *G*′ and *G*″ were measured over a range of angular frequencies (0.1–10 Hz) at 1% oscillating shear strain. The strain sensitivity (the strain for damage to occur) of various hydrogels was evaluated by conducting a strain sweep test at 1 Hz, with the oscillatory shear strain increasing from 1 to 1000%. Based on the results of the strain sweep test, the self-healing properties were assessed through damage-healing cycles involving continuous step changes in oscillatory strain at 1 Hz between 1% and n % (where n % represents the strain necessary for the gel-to-sol transition). In the static shear experiment, the steady shear viscosities of the hydrogels were measured across a range of increased shear rates (1–100 s^–1^). Thixotropic behavior of PDUB2 and PDUB2′ hydrogels was evaluated using a three-interval time sweep at 25 °C, consisting of low shear (1 s^–1^ for 120 s), high shear (60 s^–1^ for 30 s), and recovery at low shear (1 s^–1^ for 120 s).

The creep and creep recovery behavior of PDBI, PDUB2, and PDUB2′ hydrogels was evaluated at 25 °C under controlled shear stresses of 25, 50, 75, and 100 Pa. A constant stress was applied for 100 s in creep mode, followed by a 200 s recovery phase after stress removal. Time-dependent strain responses were recorded by using the rheometer operating in the shear creep mode.

### 5.4 Structural and Morphological Characterizations by SAXS Experiments and TEM

SAXS measurements for both PUB and PUB′ nanoparticles, along with time-resolved SAXS analyses of PDUB and PDUB′ hydrogels, were carried out at beamline stations 13A of the National Synchrotron Radiation Research Center (NSRRC) in Hsinchu, Taiwan. A SAXS experiment was performed to investigate the time-dependent structural characteristics of PDUB and PDUB′ hydrogels at 25 °C. The mixture of precursor and nanocross-linker solutions [PD and PUB (or PUB′)] was loaded into the instrument at time *t* = 0, with scattering intensity recorded every 4 min for a total duration of 20 min. The photon energy employed was 8 keV, and a 1 s exposure time was utilized to capture SAXS profiles. The scattering factor (*q* range) was defined within the range of 0.0028–0.28 Å^–1^. The radius of gyration (*R*_g_) was estimated using Guinier analysis under the condition *qR*_g_ < 1.3. The shape factor (*R*_g_/*R*_h_) was subsequently calculated based on the *R*_g_ value and the hydrodynamic radius (*R*_h_; which is a half of the hydrodynamic diameter, i.e., *D*_h_/2).

The scattering curves obtained from SAXS experiments were analyzed and fitted by using SasView 5.0.6 software. The following model function was applied to fit the SAXS data

1 $$I\left(q\right)=I_{p}\left(q\right)+I_{er}\left(q\right)S_{hs}\left(q\right)+I_{lrz}\left(q\right)+I_{bkg}$$

where *I*(*q*) represents the total intensity following the data reduction process applied to the experimental results. *I*_bkg_ refers to the background intensity.

*I*_P_ is the power-law model, defined as

2 $$I_{P}\left(q\right)=I_{scale}{\cdot q}^{-D_{f}}$$

where the fractal dimension (*D*_f_) characterizes the large-scale inhomogeneities within the hydrogel. *I*_scale_ represents the scaling factor.

*I*_er_(*q*) is the ellipsoid model for oriented ellipsoids defined by the following equations

3 $$I_{ep}\left(q\right)=I_{0}P\left(q,\alpha \right)=I_{0}\frac{scale}{V}F^{2}\left(q,\alpha \right)$$

4 $$F\left(q,\alpha \right)=\Delta \rho V\cdot \frac{3\left(\sin qr-qr\cos qr\right)}{{\left(qr\right)}^{3}}$$

5 $$r={[R}_{e}^{2}\sin^{2}\alpha +R_{p}^{2}\cos^{2}{\alpha ]}^{1/2}$$

where α is the angle between the axis of the ellipsoid and $\vec{q}$ , and *R*_p_ represents the polar radius along the rotational axis. *R*_e_ refers to the equatorial radius, which is perpendicular to the rotational axis; Δρ is the scattering length density difference between the scatterer and the solvent.

*S*_hs_ (*q*) is the hard-sphere structure factor to model the correlation between spherical particles interacting via hard sphere (excluded volume) interactions.

6 $$S_{hs}\left(q\right)=\frac{1}{\left[1+24\eta G\left(2R_{HS}q\right)/2R_{HS}q\right]}$$

where *R*_HS_ represents the hard-sphere radius, defined as half the center-to-center distance between particles. The parameter η denotes the hard-sphere volume fraction, indicating the proportion of particle correlation within the system. The parameter *G* is defined below

7 $$G\left(x\right)=\gamma \frac{\sin x-x\cos x}{x^{2}}+\kappa \frac{2x\sin x+\left(2-x^{2}\right)\cos x-2}{x^{3}}+\varepsilon -x^{4}\cos x+4(\frac{3x^{2}-6\cos x+\left(x^{3}-6x\sin x+6\right)}{x^{5}}$$

where γ = $\frac{{\left(1+2\eta \right)}^{2}}{{\left(1-\eta \right)}^{4}}$ , κ = $\frac{{-6\eta \left(1+\eta /2\right)}^{2}}{{\left(1-\eta \right)}^{4}}$ , and ε = γη/2.

*I*_oz_(*q*) represents the Ornstein–Zernike equation, which characterizes the spatial correlation of local chain segments within the system

8 $$I\left(q\right)=\frac{I_{scale}^{\prime }}{1+{\left(\xi L\right)}^{2}}$$

where the correlation length (ξ) represents the characteristic distance over which concentration fluctuations occur, while *I*_scale_′ is the scaling factor. These fluctuations contribute to the scattering observed in the high-*q* region of the SAXS curve.

The morphology of PUB and PUB′ nanoparticles was examined using TEM (JEM-2000FXII, JEOL, Japan). For sample preparation, PUB and PUB′ dispersions were diluted to 1000 ppm with distilled water and dropped onto copper grids, followed by a 5 min resting period. Excess liquid was then carefully removed by using filter paper. To enhance image contrast, 1 wt % phosphotungstic acid (Sigma-Aldrich, USA) was applied to the grids as a stain. The stain was left on the grid for 30 s before being blotted off, and the samples were then examined under TEM. For hydrogel samples, PDUB2 and PDUB2′ mixtures were diluted to 5000 ppm with distilled water. The mixtures were subjected to the same staining and imaging procedures as those described above.

### 5.5 Self-Healing Rate and 3D Printability of PDUB2 and PDUB2′ Hydrogels

The self-healing behavior of each PDUB and PDUB′ hydrogel was assessed by monitoring the disappearance of central holes in the hydrogel. The hydrogel was positioned on a disc with a diameter of 1.5 cm, and a circular hole with a diameter of approximately 0.5 cm was created at the center. The healing progress of the hole was recorded, and the data were quantified and analyzed. Each experiment included three samples, and each group’s experiment was conducted in triplicate to ensure reproducibility of the data.

The PDUB2 and PDUB2′ hydrogels were further tested for the 3D printability in order to serve as a sacrificial material for 3D printing. The printing of hydrogels was carried out at 25 °C with a commercial bioprinter (Regenovo, Bio-Printer-WS, China). The PDUB2 or PDUB2′ hydrogel was extruded through a cone-like nozzle (size 160 μm) by adjusting the printing pressure (0.35–0.45 MPa) and extrusion velocity (2–3 mm s^–1^). The hydrogel as ink was successfully printed through the 160 μm nozzle to create scaffolds with designed dimensions of 1.2 × 1.2 × 1.2 cm (*W* × *D* × *H*). The actual stacking height was measured to assess the stackability. After 3D printing, the printed structure was placed under a microscope to observe the diameter of the printed filaments. The extrusion expansion ratio (%) of the printed filaments was calculated as the percent change in diameter relative to the nozzle diameter. Additional 3D printing was performed using a 260 μm cone-like nozzle under the same printing conditions. A leaf and a snowflake were printed as single-layer patterns to demonstrate fine pattern resolution, while an octopus structure with 60 deposited layers was printed to evaluate the vertical stacking capability.

### 5.6 Cell Culture and Cytocompatibility for PDUB and PDUB′ Hydrogels

ECs of Sprague–Dawley rats were extracted from the carotid arteries of adult rats. ECs were cultured in the EC growth medium-2 (EGM-2; Lonza, BulletKitTM, USA) containing 1 g L^–1^ glucose. The culture medium was refreshed every two days, and cell passaging was performed upon reaching 80%–90% confluence in the cell culture. For subsequent experiments, cells from the third passage were utilized. Cells were maintained in a 37 °C incubator with 5% CO_2_.

ECs (density of 1 × 10^6^ cells mL^–1^) were uniformly blended with PDUB and PDUB′ hydrogels. The cell vitality assay employed Solution 5 (ChemoMetec, Denmark), which contained propidium iodide (PI), acridine orange (AO), and VitaBright-48TM (VB-48). ECs were combined with the EGM-2 and PDUB (or PDUB′) hydrogels (with PBS as the solvent). The evaluation of cell vitality within the hydrogel was conducted using NucleoCounter NC-3000, a full-spectrum fluorescence microscope after 1 h of incubation. The dead cells were distinguished by the red stain from PI, while AO served as the counterstain for all nuclei and imparted a green color. VB-48 imparted a blue color to viable cells based on their level of thiols.

### 5.7 Glucose Sensitivity of the PDUB2 and PDUB2′ Hydrogels

PDUB2 and PDUB2′ hydrogels were individually placed into dishes with a diameter of 3.5 cm for gelation. Subsequently, all of the hydrogels were weighed to determine their initial weight (*W*_i_). All hydrogels were then submerged in EGM-2 containing 1 g L^–1^ glucose with the proportion maintained at 1:1 relative to the hydrogel, and changes in the weight of each hydrogel were monitored at specific time intervals. After all hydrogels were lyophilized to eliminate the medium, the constructs were weighed to determine the residual weight (*W*_f_). The remaining weight (%) of each hydrogel was calculated by the equation *W*_f_/*W*_i_ × 100%. Each independent experiment comprised four samples, and each experimental group was replicated three times to ensure reproducibility of the data.

### 5.8 Preparation of Constructs with Hollow Microchannels Using the PDUB2′ Hydrogel as the Template

The nonsacrificial gelatin aqueous solution was initially cast onto a dish as the base supporting layer and maintained at the printing platform temperature of 15 °C. A sacrificial template composed of the PDUB2′ hydrogel was then fabricated onto this gelatin layer by 3D printing at 25 °C using the bioprinter through a 260 μm cone-like nozzle. Subsequently, an additional gelatin solution was cast over the entire construct to completely encapsulate all of the sacrificial templates. The embedded structure was immersed in EGM-2 containing 1 g L^–1^ glucose at 25 °C, which enabled the medium to permeate the gelatin and reach the sacrificial template. Upon dissolution of the template, hollow channels were generated in the gelatin. Channels were stained with trypan blue solution (Thermal Fisher, USA) and safranin O solution (Sigma-Aldrich, USA) to confirm the successful formation of hollow structures.

### 5.9 Statistical Analysis

All quantitative values are expressed as the mean ± standard deviation. Each individual experiment comprised multiple samples (*n* = 3). Each experimental group was replicated three or more times to ensure data reproducibility. Statistical differences between groups were analyzed by using one-way analysis of variance (one-way ANOVA). Differences were considered statistically significant when the *p* value was less than 0.05. This rigorous experimental design and statistical analysis were employed to enhance the reliability and validity of our findings.

## Supplementary Material



## Notes

Characterization of boronic acid-functionalized PU nanoparticles (PUB and PU) and their incorporation into PD-based hydrogels (PDUB1, PDUB3, and PDUB4); evaluation of control hydrogels composed of PD + borax and PD + borax + PU; and data of nanoparticle size and morphology (TEM images), gelation time, self-healing ability, rheological properties, SAXS fitting profiles, thixotropic behavior, creep recovery, cytocompatibility, and 3D printability (PDF)
