# Supplementary material for: Soft, Deformable Polyurethane-Boronic Acid Nanoparticles as Dynamic Cross-Linkers to Construct 3D-Bioprintable Hydrogels
Source: ACS Appl Mater Interfaces. 2025 Jul 8;17(30):43565–83. doi: 10.1021/acsami.5c07208 (PMC12314868; doi:10.1021/acsami.5c07208)
Supplement: Supplementary file 1 [file am5c07208_si_001.pdf]

Supporting Information

Soft, Deformable Polyurethane-Boronic Acid Nanoparticles as Dynamic Cross-linkers to Construct 3D-Bioprintable hydrogels

Yung-Chen Chang [a], *Tsai-Yu Chen* [a], *Yi-Ming Sun* [b], *Shan-hui Hsu* [a*]

[a] Institute of Polymer Science and Engineering, National Taiwan University, Taipei 106319, Taiwan, Republic of China

[b] Department of Chemical Engineering and Materials Science, Yuan Ze University, Chung-Li, Taoyuan 32003, Taiwan, Republic of China

* Shan-hui Hsu (Corresponding author)

Institute of Polymer Science and Engineering, National Taiwan University, No. 1, Sec. 4 Roosevelt Road, Taipei 106319, Taiwan, ROC.; Phone: +886-2-3366-5313; Fax: +886-2-3366-5237; E-mail: shhsu@ntu.edu.tw

Supplemental tables:3; Table S1 to Table S3

Supplemental figures:11; Figure S1 to Figure S11

Table S1. The hydrodynamic size, zeta potential, and polydispersity index (PDI) of PU nanoparticles in water suspension, along with the molecular weight (Mw) of PU chains determined in DMAc solution.

	R_h (nm)	Zeta potential (mV)	PDI	Mw
PU	14.3 ± 3.5	-40.2 ± 1.21	~0.059	91163

Table S2. The composition and gelation time of PDUB1, PDUB3, and PDUB4 hydrogels prepared from PD and PUB.

	PD (wt%)	PUB (wt%)	Borax contained in PUB (wt%)	PEI (wt%)	Gelation time (min)
PDUB1	12.2	12.7	1.14	0	~10
PDUB3	12.2	6.3	0.57	0	~20
PDUB4	12.2	4.2	0.38	0	>30

Table S3. The composition and gelation time of PD + borax and PD + borax + PU hydrogels. The PU used here refers to NH_2-capped polyurethane synthesized using ethylenediamine (EDA) as the chain extender, distinct from the borax-containing PUB and PUB' used in PDUB2 and PDUB2' formulations.

	PD (wt%)	Borax (wt%)	PU (wt%)	Gelation time (min)
PD + borax	12.2	0.76	0	~2.5
PD + borax + PU	12.2	0.76	8.4	~3

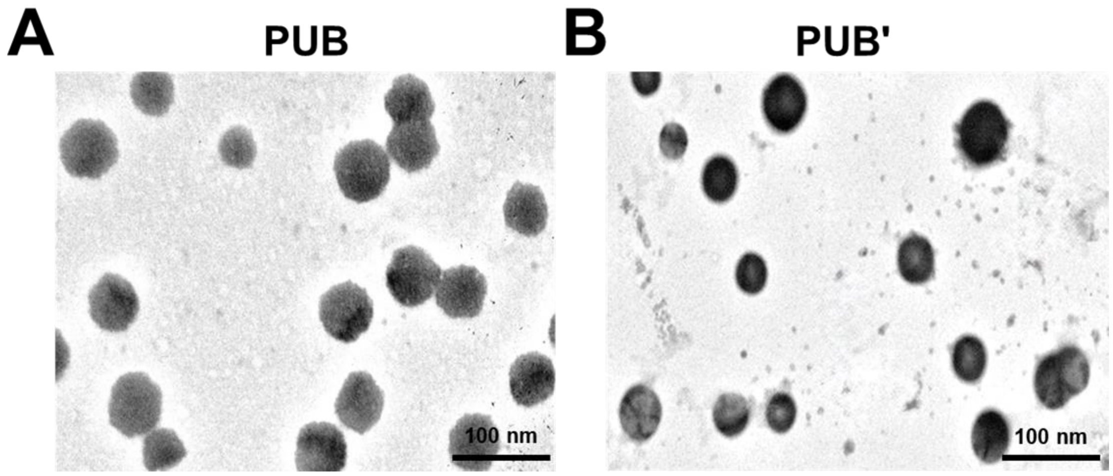

Figure S1. TEM images of PUB and PUB' nanoparticles. (A) PUB nanoparticles are well dispersed and nearly spherical. (B) PUB' nanoparticles are also well dispersed, but they display a slightly ellipsoidal morphology. Scale bars, 100 nm. The specimens were prepared from PUB and PUB' dispersions diluted to 1000 ppm with distilled water, drop-cast onto copper grids, and stained with 1 wt% phosphotungstic acid for 30 s.

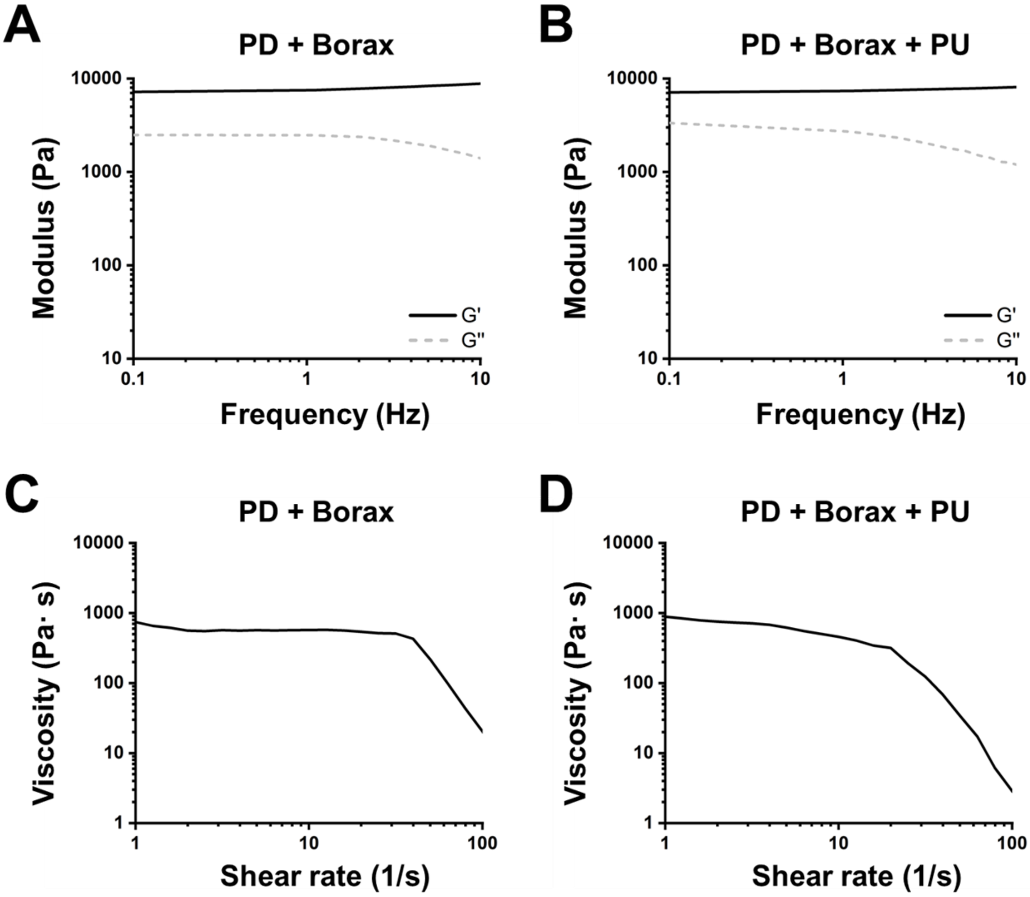

Figure S2. Rheological properties of PD + borax and PD + borax + PU hydrogels. Frequency sweep results show that both (A) PD + borax and (B) PD + borax + PU hydrogels exhibit gel-like behavior, with storage modulus (G') consistently higher than loss modulus (G") across the tested frequency range. Steady shear viscosity curves of (C) PD + borax and (D) PD + borax + PU hydrogels indicate limited shear-thinning behavior in both systems. The incorporation of PU results in only a slight improvement in shear-induced viscosity reduction, which remains insufficient for smooth extrusion through a 30G nozzle.

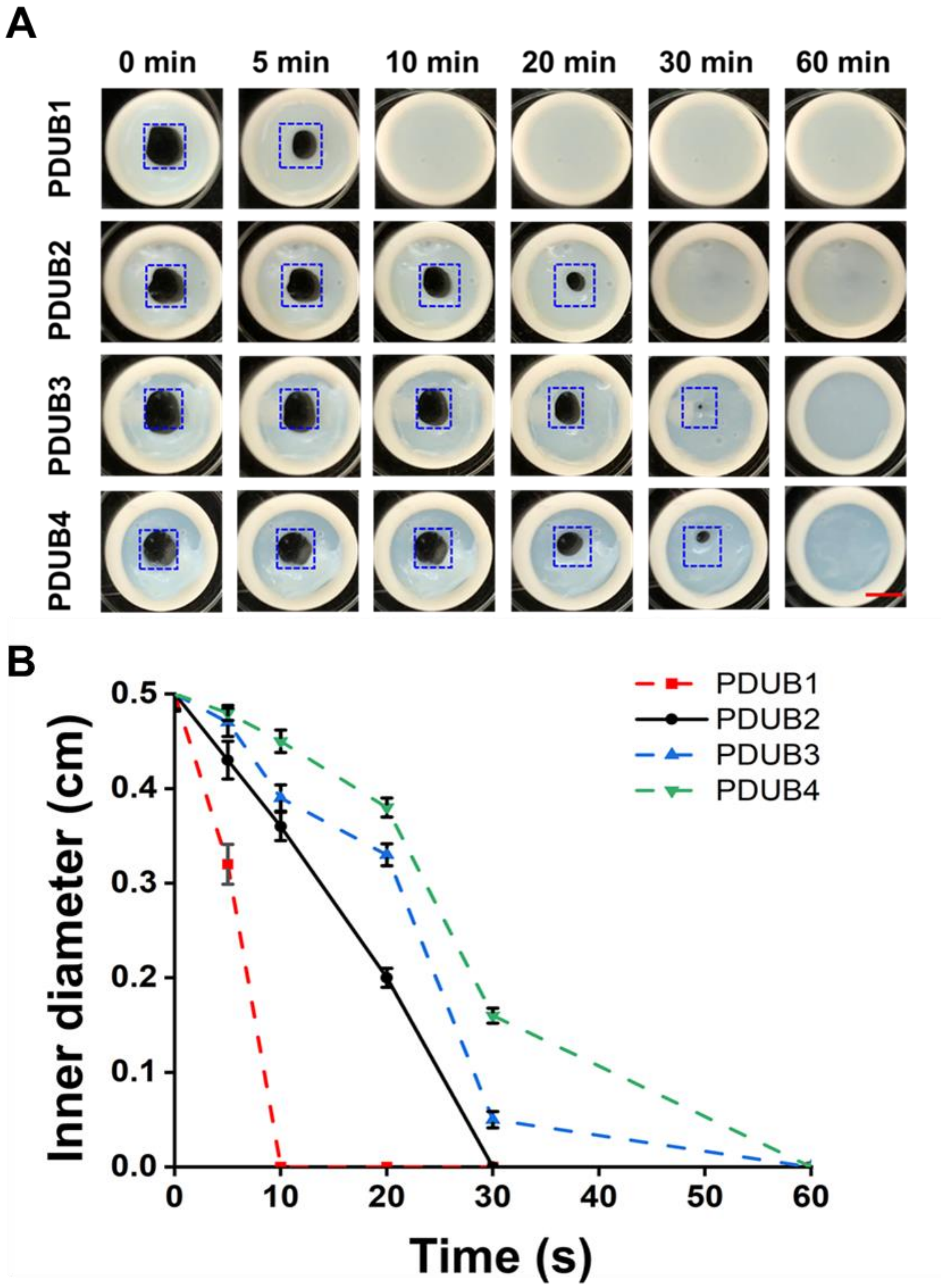

Figure S3. Self-healing ability of PDUB hydrogels. (A) Macroscopic evaluation of self-healing in PDUB hydrogels demonstrated by the closure of central holes. Scale bar, 0.5 cm. The highlighted squares in the figure mark the presence of these holes. (B) Variation in the hole diameter over time during the healing process.

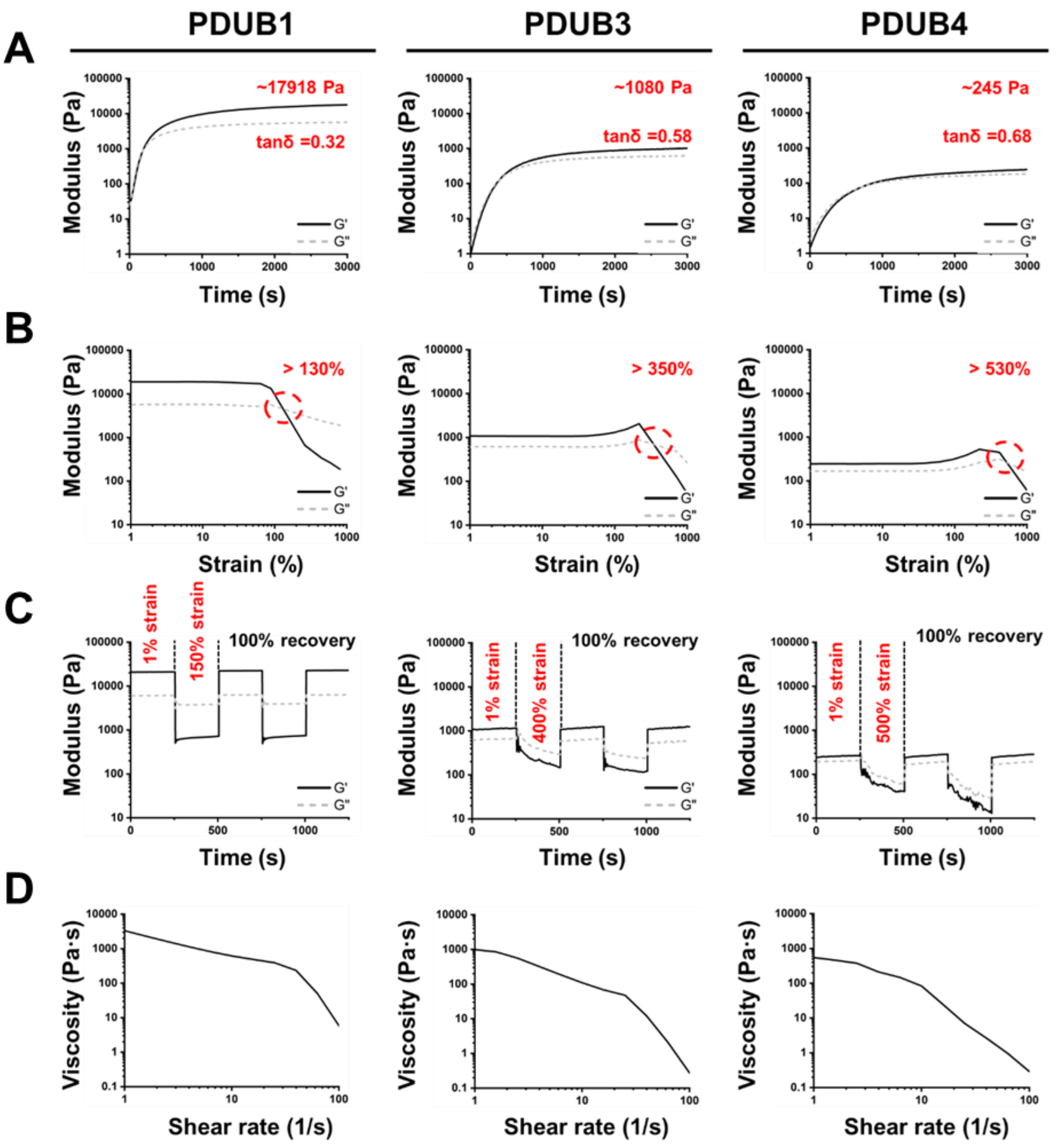

Figure S4. Rheological properties of PDUB1, PDUB3, and PDUB4 hydrogels. (A) Storage modulus (G') and loss modulus (G") of PDUB1, PDUB3, and PDUB4 hydrogels during gelation at 25 °C, measured at 1 Hz and 1% strain. (B) Strain sweep of equilibrated PDUB1, PDUB3, and PDUB4 hydrogels, measured at 1 Hz and 25 °C. (C) Self-healing experiments showing G' and G" of equilibrated PDUB1, PDUB3, and PDUB4 hydrogels at 1 Hz during alternating cycles of 1% strain and damaging strains (%) of each PDUB hydrogel. (D) Steady shear viscosities of PDUB1, PDUB3, and PDUB4 hydrogels measured at 25 °C over a shear rate range of 1-100 s^{-1}.

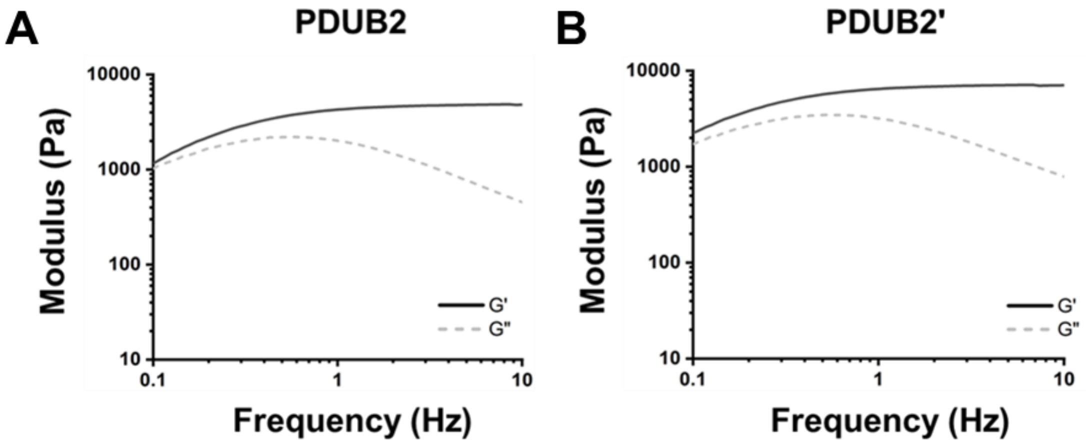

Figure S5. Frequency sweep for PDUB2 and PDUB2' hydrogels. The dynamic frequency-dependent rheological properties of (A) PDUB2 hydrogel and (B) PDUB2' hydrogel were measured at an oscillatory strain of $\gamma = 1\%$. Both hydrogels show G' > G'' in the whole frequency range. Beyond ~1 Hz frequency, G' was stabilized while G'' declined.

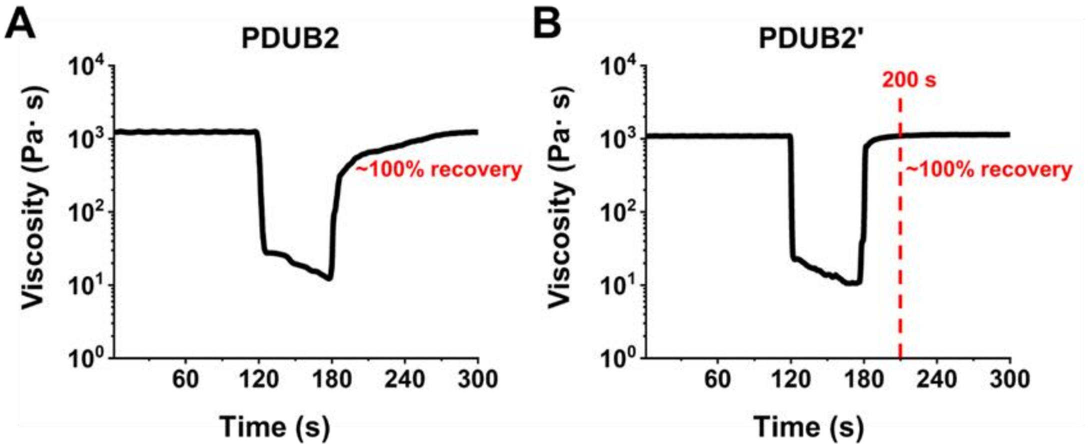

Figure S6. Thixotropic behavior of PDUB2 and PDUB2' hydrogels assessed using a three-interval time test (1 s^{-1} for 120 s, 60 s^{-1} for 60 s, and 1 s^{-1} for 120 s) at 25 °C. (A) The PDUB2 hydrogel exhibited gradual viscosity recovery and reached equilibrium at approximately 300 s. (B) The PDUB2' hydrogel displayed faster recovery, with viscosity returning to baseline levels by around 200 s.

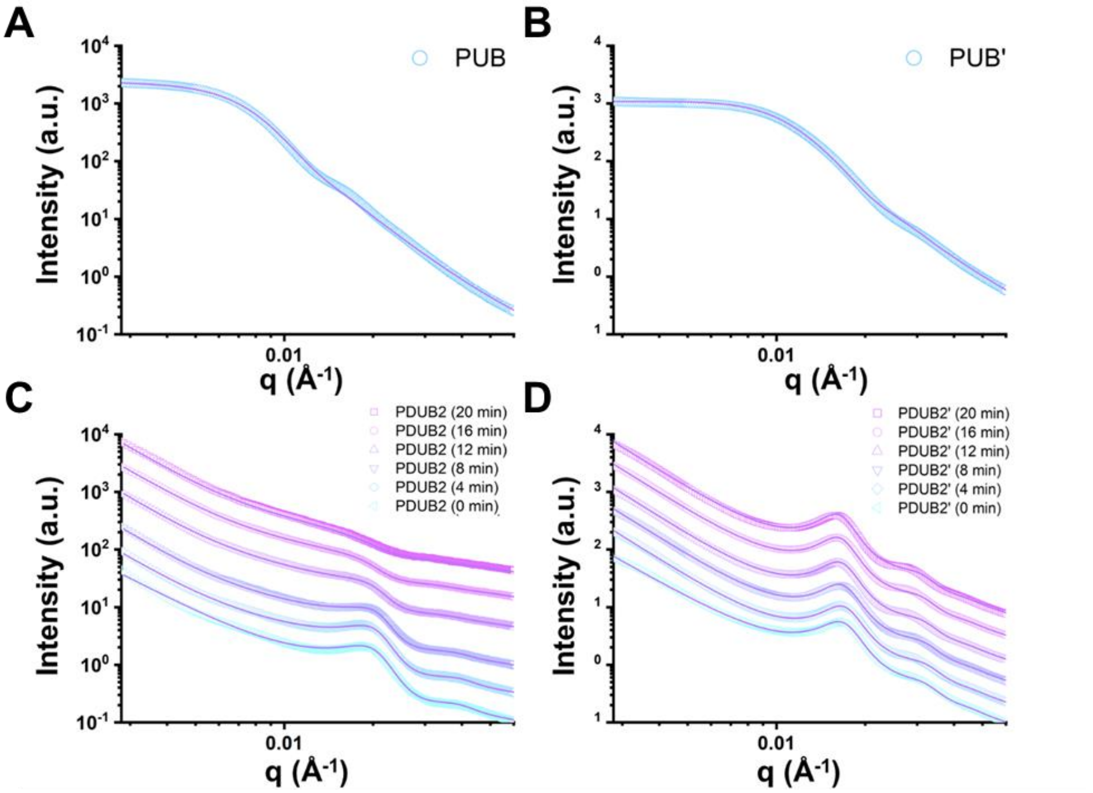

Figure S7. The SAXS profiles and fitting results for (A) PUB nanoparticles, (B) PUB nanoparticles, (C) PDUB2 hydrogel, and (D) PDUB2' hydrogel. Data for (C) and (D) were collected at 4-minute intervals during the experiment.

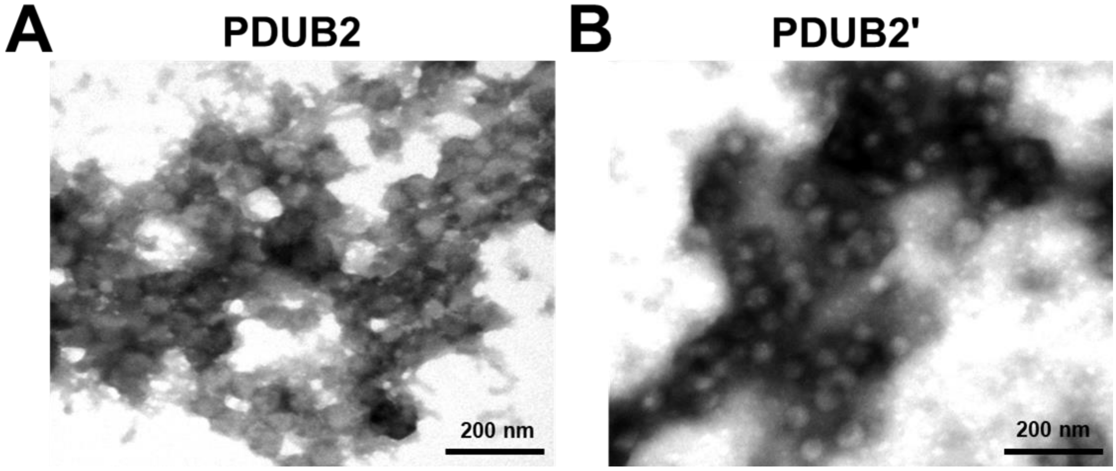

Figure S8. TEM images of PDUB2 and PDUB2' hydrogels. (A) PUB nanoparticles within PDUB2 hydrogel lose their spherical outline and are flattened into irregular aggregates. (B) PUB' nanoparticles within PDUB2' hydrogel retain the ellipsoidal morphology and are densely distributed in the hydrogel matrix. Scale bars, 200 nm. Specimens were prepared from PDUB2 and PDUB2' mixtures diluted to 5000 ppm with distilled water, drop-cast onto copper grids, and stained with 1 wt% phosphotungstic acid for 30 s.

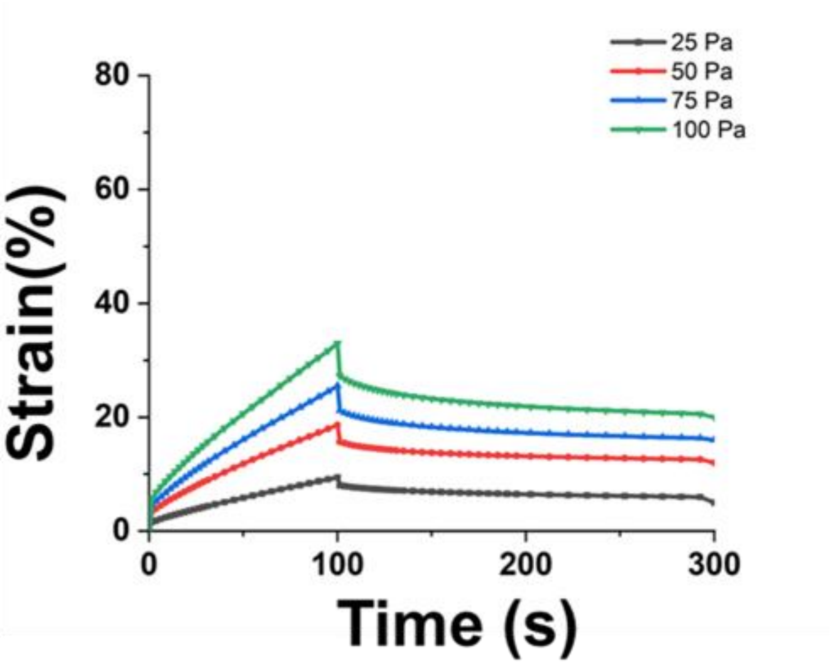

Figure S9. Creep and creep recovery behavior of PDBI hydrogel under applied stresses of 25, 50, 75, and 100 Pa at 25 °C. There is an immediate deformation when stress is applied in different levels, as well as an immediate partial strain recovery upon stress removal.

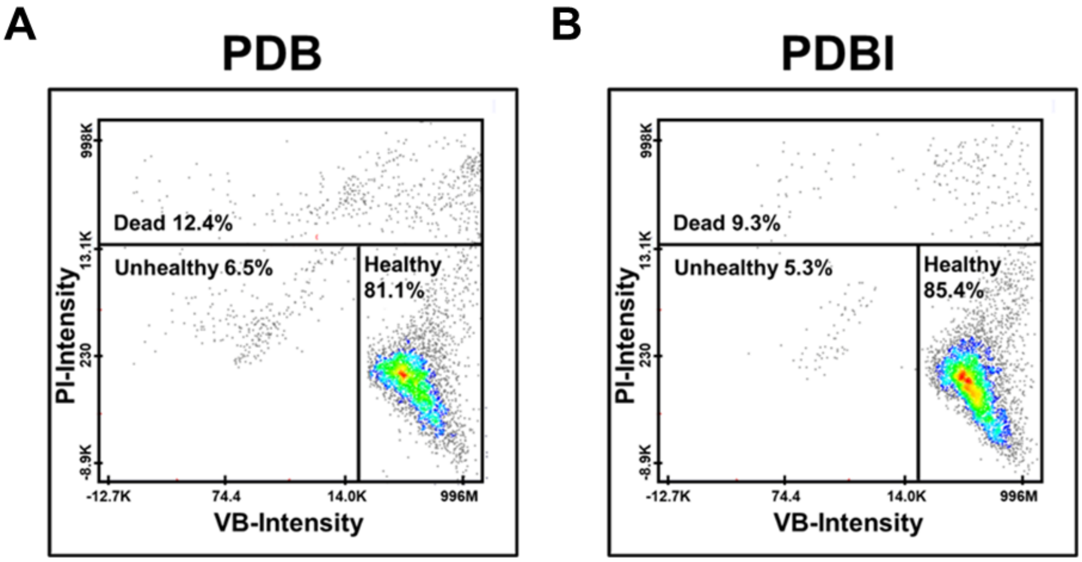

Figure S10. Cytocompatibility evaluation of ECs encapsulated in (A) PDB and (B) PDBI hydrogels. VB-48 staining was used to distinguish healthy, unhealthy, and dead cell populations.

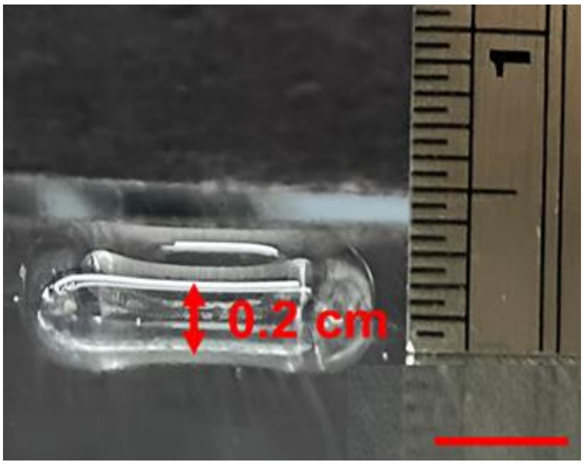

Figure S11. The side view of PDBI hydrogel after printing. Scale bar, 0.5 cm.
